# MIRTH: Metabolite Imputation via Rank-Transformation and Harmonization

**DOI:** 10.1186/s13059-022-02738-3

**Published:** 2022-09-01

**Authors:** Benjamin A. Freeman, Sophie Jaro, Tricia Park, Sam Keene, Wesley Tansey, Ed Reznik

**Affiliations:** 1 Computational Oncology, Department of Epidemiology and Biostatistics, Memorial Sloan Kettering Cancer Center, New York, USA; 2 Electrical Engineering Department, The Cooper Union, New York, USA

**Keywords:** Metabolomics, Missing data, Imputation, Unmeasured metabolites, Matrix factorization

## Abstract

Out of the thousands of metabolites in a given specimen, most metabolomics experiments measure only hundreds, with poor overlap across experimental platforms. Here, we describe Metabolite Imputation via Rank-Transformation and Harmonization (MIRTH), a method to impute unmeasured metabolite abundances by jointly modeling metabolite covariation across datasets which have heterogeneous coverage of metabolite features. MIRTH successfully recovers masked metabolite abundances both within single datasets and across multiple, independently-profiled datasets. MIRTH demonstrates that latent information about otherwise unmeasured metabolites is embedded within existing metabolomics data, and can be used to generate novel hypotheses and simplify existing metabolomic workflows.

**Supplementary Information:**

The online version contains supplementary material available at 10.1186/s13059-022-02738-3.

## Background

Large-scale quantification of metabolite pool sizes (“metabolomics”) is a powerful approach for the mechanistic investigation of metabolic pathway activity and the identification of metabolic biomarkers of disease and therapeutic response [1–5]. By observing how metabolite levels are altered in various physiological conditions, metabolomics can reveal the role of metabolites in homeostasis, in disease, or in response to perturbations [6].

The bulk of large-scale metabolomics data in biology research is now generated using mass spectrometry [7]. This technology ultimately reports the number of measured ions associated with a unique metabolite in a given biological specimen. To accurately identify metabolites, targeted metabolomics studies must be calibrated for maximum sensitivity for specific classes of metabolites with similar chemical properties [8]. Consequently, each metabolomics platform can only measure a subset of the entire assortment of metabolites in a specimen. Metabolomics assays operated in different laboratories often measure sets of metabolites with little overlap. For example, in a pan-cancer series of eleven metabolomics datasets [9], only 23 out of 935 metabolites were measured across all samples. This lack of overlap restricts cross-dataset comparisons and impedes the discovery of general principles of metabolite regulation across datasets. The goal of this work is to enable cross-dataset comparisons by developing a method to impute missing metabolites between datasets.

Imputing missing values is specifically challenging in metabolomic data analysis because metabolite levels are reported in arbitrary units, which we refer to as relative abundance. A relative abundance level only contains information about the concentration of a metabolite in a sample relative to all other measurements of that metabolite in that dataset. These levels are not comparable between different metabolites in the same dataset, nor are they comparable to the measurements of the same metabolite in different datasets. The lack of a shared measurement scale between metabolites and datasets prevents the application of existing imputation methods that assume a common basis (e.g., probabilistic PCA [10]). Others have developed methods for the imputation of single metabolomics datasets, including some based on k-nearest neighbor imputation [11, 12], quantile regression imputation of left-censored data and random forest imputation [13], kernel-weighted least squares imputation [14], and multivariate imputation by chained equations [12]. These methods impute left-censored values—missing values arising when a metabolite level falls below a detection threshold in a subset of samples—within a single dataset [13].

**Fig. 1:**
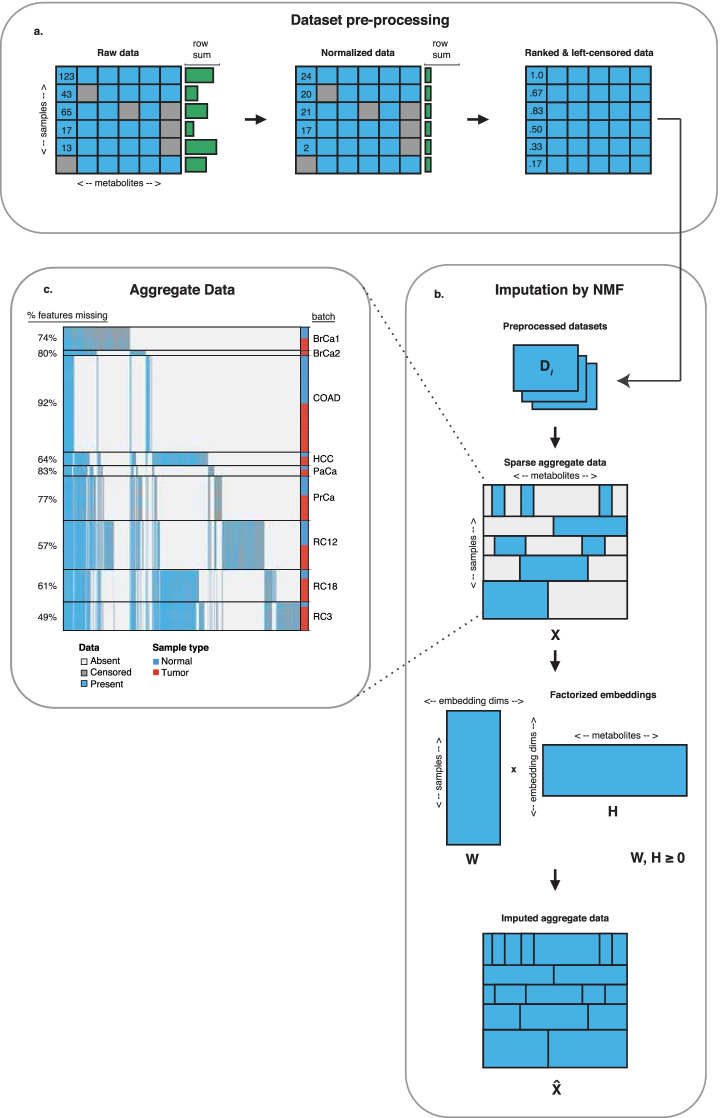
Workflow for MIRTH Imputation of Metabolomics Data. **a** Individual datasets are normalized and rank-tranformed, accounting for left-censoring. **b** Preprocessed datasets ($\varvec{D_i}$) are combined into a sparse aggregate data matrix ($\varvec{X}$), which is then factorized into embedding matrices $\varvec{W}$ and $\varvec{H}$. The product $\varvec{WH}$ yields an imputed data matrix ($\varvec{\hat{X}}$). **c** Aggregate data from 9 pan-cancer metabolomics datasets with tumor and normal samples reveals poor across-dataset metabolite feature overlap and high degree of missingness

In contrast to the above-mentioned work, we consider here a related but larger and more challenging class of problems related to imputing entirely-unmeasured metabolite features across datasets. We present Metabolite Imputation via Rank-Transformation and Harmonization (MIRTH), a relative abundance matrix factorization model that learns relationships between metabolite levels in one or more metabolomics datasets. MIRTH’s key insight is that transforming relative abundance levels to normalized ranks maps every measurement to a comparable scale between metabolites and across batches. Critically, rank transformation enables MIRTH to discover patterns of covariation between metabolite pools that are shared across datasets without making assumptions about the relative concentrations of the same metabolite across datasets. MIRTH factorizes rank-transformed metabolomics data into two low-dimensional embedding matrices (Fig. 1). These embeddings describe the latent structure between samples and metabolite features. By compressing the information contained in the space of all metabolite features measured across all datasets into low-dimensional embeddings, MIRTH discovers correlative relationships among metabolites across datasets. These correlations enable the imputation of unmeasured features in each dataset. Matrix factorization has been applied to imputing metabolomics data [15], but MIRTH differs from this previous work in two key respects. First, MIRTH makes use of cross-validation rather than any potentially-biased priors to tune the number of embedding dimensions. Second, MIRTH addresses the imputation of entirely missing metabolites across datasets, enabled by rank-transformation, while previous work imputes only left-censored values in one dataset. Matrix factorization techniques have also previously been applied to a variety of other data modalities  [16], including gene expression data [17–19], protein sequences [19], and genomic data [20] for clustering analysis and class discovery.

We evaluate the performance of MIRTH in a pan-cancer series of nine metabolomics datasets. MIRTH achieves high accuracy in in silico experiments predicting ranks. In each of our nine batches of experimental data, a proportion of simulated-missing metabolites entirely masked from a batch are imputed well by MIRTH. In kidney cancer data with paired tumor and normal tissues, MIRTH correctly predicts unmeasured tumor-enriched and tumor-depleted metabolites in one dataset by transferring information from a second dataset where those metabolites were measured. MIRTH also accurately imputes metabolites across ionization modes, enabling the imputation of unmeasured metabolites across chemically distinct classes. By increasing the available information about the metabolome, MIRTH increases the hypothesis-generating potential of existing datasets while revealing new information embedded in existing metabolomics data.

## Results

We completed a series of benchmark studies to assess the performance of MIRTH in different imputation tasks. We evaluated the performance of MIRTH on a collection of nine previously-published mass-spectrometry metabolomics datasets, consisting of original raw ion counts before any preprocessing, e.g., normalization and imputation [9]. Metabolite names were harmonized by maximizing consistency across multiple metabolite identifiers, as previously described [9]. Details on the number of samples and features in each dataset are reported in Additional file 1: Table S1.

For each benchmark, we measured the concordance between the true and imputed ranks of metabolite samples in held-out data. Across experiments, we found MIRTH performed well in high sample-to-metabolite scenarios. In contrast, MIRTH performed poorly when there were insufficient samples to train on and when samples were highly censored. Furthermore, we found that a subset of metabolites were reproducibly well-imputed across different datasets and imputation tasks, ascribing a quantitative metric of confidence to MIRTH’s predictions.

### MIRTH recovers missing metabolites within metabolomics datasets

**Fig. 2:**
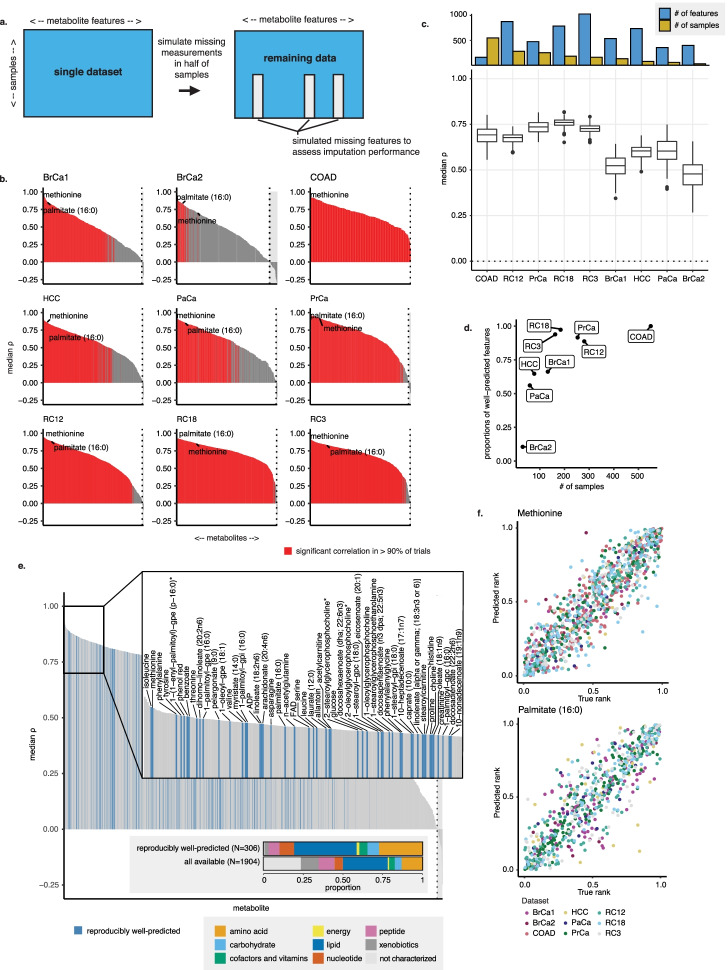
MIRTH achieves high accuracy imputing within datasets. **a** Samples for a subset of features were masked in half of all samples in a dataset before imputation to create data on which to assess imputation performance. **b** Imputation performance by dataset is reported by median $\rho$ values across all simulated-missing features in each MIRTH iteration. **c** Imputation performance by metabolite, reported as the median $\rho$ value for each metabolite across all trials, is plotted for each batch. Metabolites are ordered by decreasing imputation performance. **d** As dataset size (number of samples) increases along the *x*-axis, the proportion of well-predicted metabolites in a dataset increases as well. This illustrates the relationship between the number of training samples and overall imputation performance. **e** Imputation performance for each metabolite summarized across datasets (median $\rho$ values across datasets are plotted). A subset of consistently well-imputed metabolites are labeled. Reproducibly well-predicted metabolites are indicated in blue. **f** The predicted ranks versus the true ranks of example metabolites, methionine and palmitate (16:0), when imputed in each single dataset. Each point represents one sample in which the metabolite was measured

We first verified that MIRTH accurately imputed missing measurements within a single dataset. This represented the most straightforward imputation task because there were no batch effects associated with merging of data from two or more distinct datasets. We performed an in silico experiment, simulating a scenario where a set of metabolites was not measured in a subset of samples in the dataset. First, we randomly select 50% of the samples to serve as hold-out samples. Next, we randomly selected 10% of all the metabolites to serve as hold-out metabolites (Fig. 2a). We masked the hold-out metabolites in the hold-out samples to simulate that they were not measured in half the dataset. This effectively split the dataset into two pseudo-datasets, where a smaller set of features was measured in the hold-out pseudo-dataset.

For each of the 9 benchmarking datasets (Additional file 1: Table S1), we split the data as described above and applied MIRTH to impute the held-out values (Fig. 2a and “Methods”). Since the held-out values in the dataset were actually known, the performance of MIRTH was assessable by comparing the actual and predicted ranks in the simulated-missing features. We repeated this experiment 200 times for each of the 9 datasets, randomly selecting a set of metabolites in a random set of samples to mask in each iteration. The optimal number of embedding dimensions, chosen through cross-validation separately for each dataset, ranged between 3 and 37 (Additional file 2: Fig. S1a). We also evaluated the performance of MIRTH and its sensitivity to dataset size and feature missingness using simulated metabolomics data. The method and results of testing single-set performance on simulated data are available in the Supplementary Information section.

When 10% of features were simulated missing in each trial, MIRTH successfully predicted the abundance of approximately 89% of metabolites (significant positive correlation with the true ranks in at least 90% of trials, $\rho >0, q<0.05$, BH-corrected, Fig. 2b). This proportion ranged from 11% in BrCa2 (likely due to its small number of samples) to 100% in COAD (Additional file 1: Table S2). Variation in MIRTH performance across datasets was partly explained by dataset size: median imputation performance was better for the five datasets with the most samples, compared to the other four (Fig. 2c). Using the same metric of predictive success, the proportion of well-predicted metabolites in each dataset likewise increased as the sample size of the dataset increased (Fig. 2d). This suggested that poor predictions may be the result of an insufficient quantity of data from which MIRTH could learn. Similarly, when the proportion of features simulated as missing was increased, imputation performance worsened (Additional file 2: Fig. S1b). This result was expected since higher proportions of masked metabolites leave less data from which MIRTH can learn. We also investigated factors associated with each metabolite feature which might influence imputation accuracy. We observed that metabolite features with larger coefficients of variation (in the raw, non-rank-transformed data) tended to have lower imputation accuracy (Additional file 2: Fig. S1c). Similarly, predictions of features with a greater number of censored measurements (i.e., those where more measurements were below the detection threshold) scored lower. This likely arose from a poor correlation between censored values’ tied-for-last ranks at the input and their uniformly-mapped ranks at the output (Additional file 2: Fig. S1d).

A total of 306 metabolites were reproducibly well-predicted by MIRTH across multiple datasets, meaning that they were measured in at least 4 datasets and well-predicted in at least three-quarters of the datasets in which they were measured (Fig. 2e). Among these well-predicted metabolites were 84 amino acids, 22 carbohydrates, 16 cofactors and vitamins, 5 energy carriers, 120 lipids, 28 nucleotides, 21 peptides, 8 xenobiotics, and 2 uncharacterized metabolites. Well-predicted metabolites were enriched in specific metabolite classes, including dipeptides, proteinogenic amino acids and various lipid subsets (Additional file 1: Table S3). For example, palmitate and methionine, which were measured in 8 and 9 datasets respectively, were both well-predicted in 8 experiments (Fig. 2f). While these benchmarking experiments were conducted in a setting where the ground truth was known, the observation that certain metabolites were reproducibly well-imputed in different settings suggests that imputation of their abundance by MIRTH in settings where the true abundance is unknown should be associated with additional confidence relative to all other metabolites. Furthermore, the ability of MIRTH to recover ranks of subsets of samples of metabolites in a single dataset motivated the use of MIRTH to impute entirely missing metabolites in a single dataset by learning from a matrix of aggregate datasets.

### MIRTH recovers missing metabolites by transferring knowledge across datasets

**Fig. 3:**
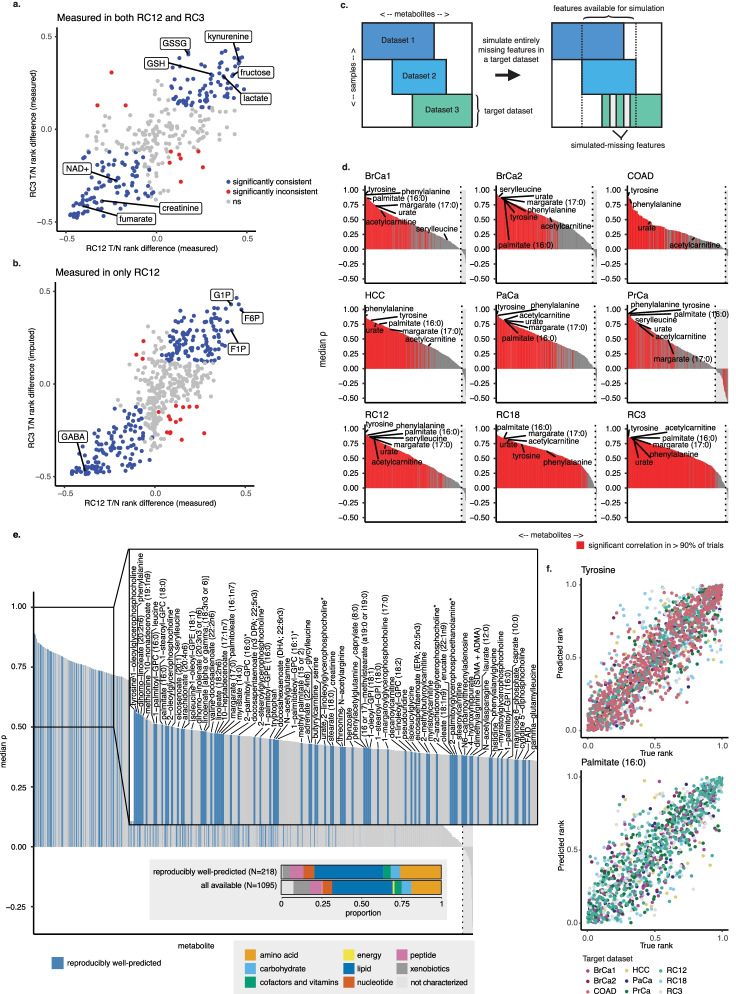
MIRTH achieves high accuracy in cross-dataset imputation and preserves biological characteristics in the data. **a** The same metabolites distinguish tumor and normal samples in RC12 and RC3. **b** Tumor-distinguishing metabolite patterns are recovered by MIRTH imputation of RC3 and RC12. **c** Schematic of experiment to assess the imputation of features that were entirely unmeasured in a dataset. **d** Typical by-metabolite $\rho$ values when those metabolite features are entirely masked from a target dataset. Metabolites are ordered by decreasing imputation performance. **e** By-metabolite performance summarized across target datasets, showing that many of the same metabolites are well-predicted in many target datasets. **f** Relationship between actual and predicted within-dataset metabolite ranks for two reproducibly well-predicted metabolites. Each point represents one sample in which the metabolite was measured

To determine if imputation of entirely unmeasured features could produce biologically sound predictions of missing metabolites, we applied MIRTH to two independent kidney cancer datasets, RC12 and RC3, each consisting of both tumor and adjacent normal tissue samples. We reasoned that metabolites distinguishing tumor and normal tissues should be highly concordant across both datasets. To test this assumption, we calculated the differential abundance of all metabolites (tumor vs. normal) in RC12 and RC3 (Additional file 2: Fig. S2). Of the 169 metabolites measured in both RC12 and RC3 that showed statistically significant differences between tumor and normal in both datasets, we observed that 159 (94$\%$) showed identical changes, including canonical metabolites such as glutathione (GSSG and GSH), lactate, NAD+, and fructose ($q<0.05$, Wilcoxon test, Fig. 3a). This demonstrated that the metabolite differences between tumor and normal samples in these datasets were comparable. Next, we repeated the analysis above considering metabolites measured in RC12, but entirely *unmeasured* in RC3, which we called *test* metabolites. After joint imputation of RC12 and RC3 with MIRTH, we compared the differential abundance of test metabolites in RC12 (where they were measured) and RC3 (where their true abundance is unmeasured, but where they have been imputed by MIRTH, Additional file 2: Fig. S2). Doing so, we again observed a strong correlation between differential abundance (tumor vs. normal) in test metabolites in RC12 and RC3. Out of 252 test metabolites, there were 235 (93%) significant and consistently differentially abundant metabolites, including glucose-1-phosphate (G1P), fructose-6-phosphate (F6P), fructose-1-phosphate (F1P), and gamma-aminobutyric acid (GABA). Only 17 metabolites inconsistently distinguished tumor and normal samples ($q<0.05$, Wilcoxon test, Fig. 3b). This analysis confirms that MIRTH preserves relationships between sample types and biologically important metabolites when imputing data across datasets, and suggests that MIRTH can be successfully applied to impute the ranks of entirely unmeasured metabolites in metabolomic data.

To further assess the ability of MIRTH to accurately impute missing features across datasets, we designated one of the nine datasets under analysis as the *target*, from which we completely masked a set of features to simulate as unmeasured. MIRTH was then applied to impute these unmeasured features, using data from the remaining eight datasets (and therefore testing the performance of MIRTH in the presence of a dataset-specific batch effect). We conducted nine such experiments, where each dataset was the *target* for one experiment (Fig. 3c). We repeated each experiment for 200 trials for each target dataset, randomly selecting 10% of features to simulate as missing each time. The optimal number of embedding dimensions ranged between 26 and 48, but equivalent-to-optimal performance could be achieved with approximately 30 dimensions (Additional file 2: Fig. S3a). Performance was evaluated similarly to the within-dataset imputation, comparing imputed and true ranks of the features simulated as missing.

Across the nine datasets under analysis, between 38% and 85% of the simulated-missing metabolites entirely masked from a target dataset were well-predicted with the MIRTH approach ($\rho >0, q<0.05$ in >90% of trials, Fig. 3d, Additional file 1: Table S4). Similar to the within-dataset imputation, performance degraded as a larger proportion of features was simulated as missing (Additional file 2: Fig. S3b). Properties of the raw data, such as the variance of the feature in the target dataset or the number of samples in other datasets where the feature was measured, partially explained why some features were better predicted than others (Additional file 2: Figs. S3c,d). Similar to the within-set imputation, MIRTH reliably predicted the ranks of certain metabolites regardless of the target dataset (Fig. 3e). There were 218 reproducibly well-predicted metabolites, consisting of 56 amino acids, 13 carbohydrates, 10 cofactors and vitamins, 94 lipids, 15 nucleotides, 18 peptides, 10 xenobiotics, and 2 uncharacterized metabolites (Fig. 3e, Additional file 1: Table S5). Similar to the within-dataset imputation, reproducibly well-predicted metabolites were enriched for lipid subsets and amino acids (Fig. 3e). Tyrosine and palmitate, for instance, were reproducibly well-predicted with a median $\rho$ of 0.892 and 0.845 respectively (Fig. 3f). These results outline a set of metabolites that are likely to be reliably imputed in a new target dataset if one were to be added to our existing aggregate set.

We also evaluated MIRTH’s across-set imputation performance on simulated metabolomics data and on data from the Cancer Cell Line Encyclopedia (CCLE) [21], the full results of which can be found in the Supplementary Information section. To summarize, applying MIRTH to the CCLE dataset resulted in comparable or better imputation of missing metabolites than with our 9 benchmarking datasets. For within- and across-dataset treatments, 100% and, typically, 94.2% of metabolites were well-predicted.

### MIRTH embeddings separate tissue of origin and metabolite class

MIRTH involves a matrix factorization that associates both metabolite features and samples with a small number of embedding dimensions. In other contexts, analysis of features and samples in embedding space can be used to interpret the similarity between samples or the covariation of different features. We therefore applied MIRTH jointly to all data available, factorizing the complete aggregate set ($\varvec{X}$) of all nine datasets. The optimal number of dimensions for the factorization of the aggregate data matrix into embedding matrices $\varvec{W}$ and $\varvec{H}$ was 30 (Additional file 2: Fig. S4a). Weights were mostly small and right-skewed (Additional file 2: Fig. S4b). We used UMAP to visualize the sample and feature embedding spaces [22].

**Fig. 4:**
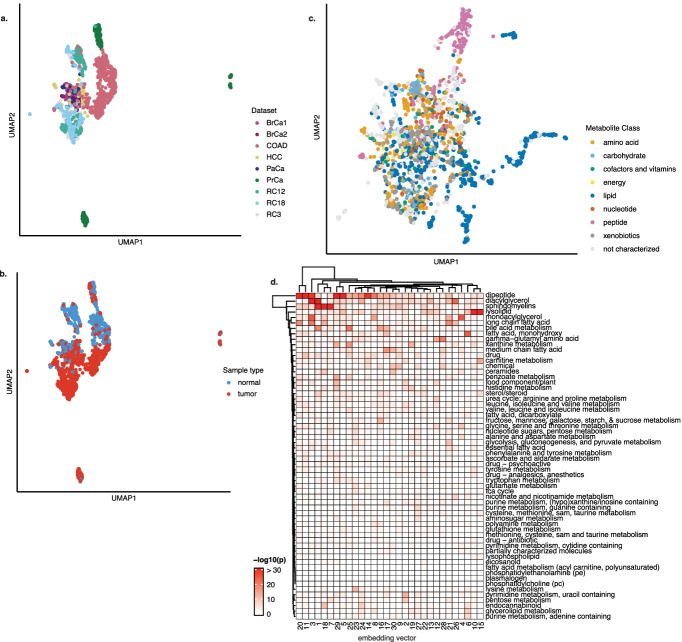
Embedding matrices reveal separation of features and samples and enrichment in certain metabolic pathways. **a** UMAP plots of sample embedding matrix **W** reveal some separation between batches and cancer types, as well as **b** separation between tumor and normal samples. **c** Feature embeddings separate peptides and lipids from other metabolites. **d** Certain pathways are enriched in certain embedding dimensions, though analysis is limited by statistical power

In sample embedding space, some clustering occurred by tissue of origin, with samples from the three kidney cancer datasets, RC12, RC18, and RC3, overlapping (Fig. 4a). COAD samples also separated from other tissues of origin. Interestingly, PrCa samples separated into three distinct clusters, raising the possibility of a latent batch effect, i.e., that the PrCa dataset consists of three sub-datasets that are not preprocessed individually by MIRTH. Definition between other tissue types, *i.e.* between BrCa1 & BrCa2, PaCa, and HCC, was less discernible. Tumor and normal samples from the same dataset also separated in embedding space along UMAP axis 2 (Fig. 4b).

Dimensionality reduction of the feature embedding matrix also revealed separation between certain metabolite classes, in particular of peptides and lipids from the rest of the measured features (Fig. 4c). The outlying peptide features predominantly represented dipeptides (Additional file 2: Fig. S4c). To determine whether individual embedding vectors were associated with functionally-related groups of metabolites, we performed a Fisher’s exact test for enrichment of a given metabolic pathway in each embedding vector after setting a cutoff value above which a feature was considered to be appreciably weighted (here, $weight=0.2$). This analysis was limited by statistical power, due to the relatively small number of metabolites in each annotated pathway. Nevertheless, the analysis identified enrichment of certain metabolite classes across multiple embedding dimensions, including dipeptides, sphingomyleins, diacylglycerols, and lysolipids (Fig. 4d).

### MIRTH imputes metabolites across MS ionization modes

Mass spectrometry-based metabolomics can be conducted in positive or negative ionization modes, which allows for quantification of metabolites that are more amenable to acquiring a positive or negative charge  [23–25]. We devised an experiment to assess the viability of predicting positive-mode measurements from negative-mode ones (or vice-versa), using a dataset where samples were profiled in both modes [24].

**Fig. 5:**
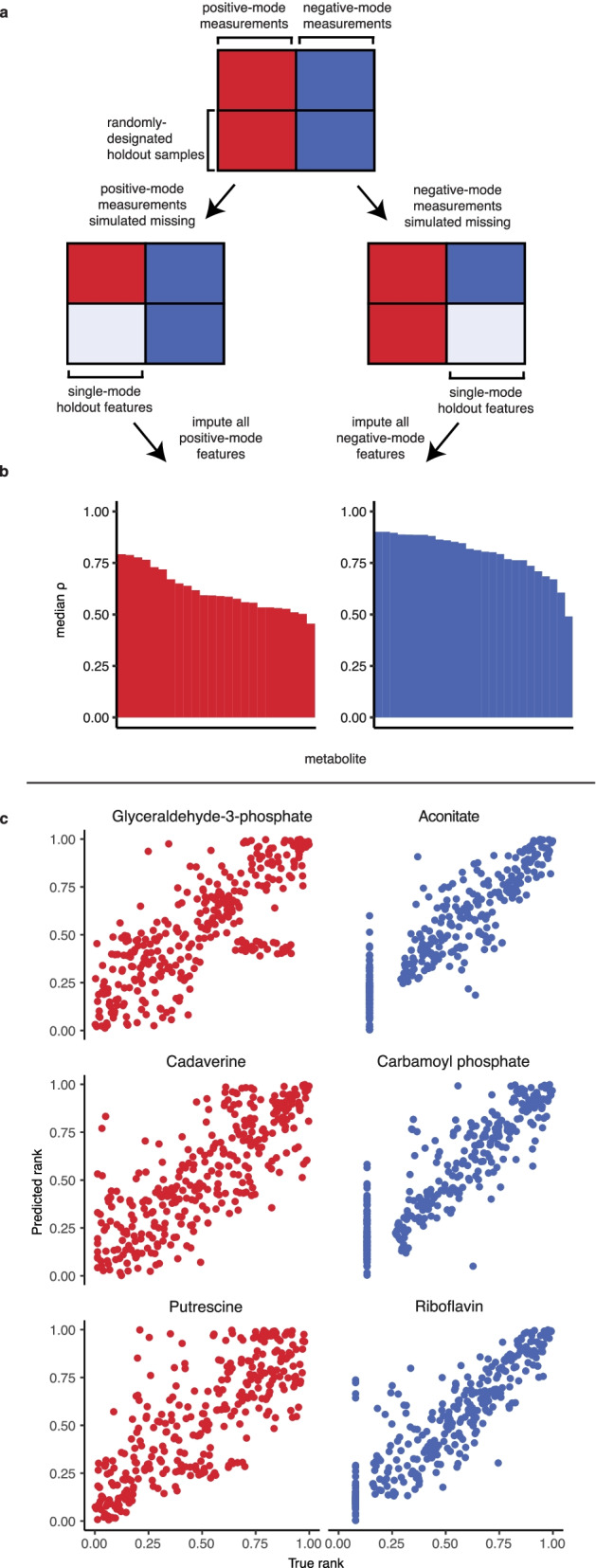
MIRTH accurately imputes across features measured in different mass spectrometer ionization modes. **a** Simulating a subset of samples as only measured in a single ionization mode, then imputing to assess performance. **b** Summarized imputation performance by ionization mode. Only metabolites measured in a single ionization mode are shown. **c** Examples of metabolites that are well-predicted across ionization modes. Each point represents one sample where the metabolite was measured

This dataset consisted of 448 features across 638 samples. Of the 241 quantified metabolites, 191 were measured in both positive and negative modes (accounting for 398 features due to redundancies). Of the remaining metabolites, 24 were measured only in positive mode and 16 were measured only in negative mode. We devised a test scenario for MIRTH whereby positive- or negative-mode measurements were completely masked from half the samples. This simulated a scenario where half of the samples were measured in both modes and the remaining samples were measured in just one mode (Fig. 5a). Imputation performance was assessed on metabolites only measured in one mode across 200 trials with a different set of samples chosen for masking each time. All non-overlapping metabolites were well-predicted ($\rho >0, q<0.05$ in $> 90\%$ of trials). Overall, negative-mode features were predicted with a higher $\rho$ than positive-mode features (Fig. 5b), perhaps due to the greater reproducibility of the positive-mode measurements on which negative-mode predictions were based [24]. Predictions for glyceraldehyde-3-phosphate, cadaverine, and putrescine - features measured only in positive mode - were notably accurate, with median $\rho$ values of 0.765, 0.788, and 0.777, respectively (Fig. 5c, Additional file 1: Table S6). Likewise, aconitate, carbamoyl phosphate, and riboflavin were among the best-predicted negative-mode features, with median $\rho$ values of 0.901, 0.888, and 0.887, respectively (Fig. 5c, Additional file 1: Table S6). Once again, these results indicate that MIRTH can impute the ranks of metabolites that were entirely unmeasured by leveraging latent information in metabolomics data—in this case, in one ionization mode.

## Discussion

MIRTH is a novel method to impute the ranks of otherwise unmeasured metabolites in semi-quantitative mass spectrometry metabolomics data by applying a matrix factorization approach tolerant to missing data. MIRTH successfully imputed ($\rho >0, p < 0.05$ in >90% of trials) between 38% and 85% of the missing metabolites in each dataset we tested. Although not all metabolites were well-predicted in all datasets, the existence of a subset of metabolite features that were reproducibly well-imputed across datasets reveals the promise of MIRTH for filling-in missing metabolites in new datasets.

That MIRTH imputes some metabolites poorly may partially be accounted for by the high variance of those features across a dataset’s samples. Nevertheless, MIRTH reliably imputes metabolites whose significant variation depends on the biological context, i.e., biologically-relevant metabolites between tumor and normal samples. This demonstration adds confidence to MIRTH predictions for metabolites whose level depends on biological context, provided that there are enough training samples across a diversity of contexts. Furthermore, the number of datasets the metabolite appeared in and the extent to which the metabolite was left-censored in each dataset affected the imputation performance on certain metabolites. These circumstances both create situations in which there is little information on which the model can train for that feature. Furthermore, there may well be technical factors that explain variation in performance across datasets. COAD samples, which yielded the best performance in within-dataset imputation, were profiled in an independent mass-spectrometry lab on a platform measuring comparatively few lipids. The PrCa dataset, which yielded the worst across-dataset performance, consisted of three subgroups of data with apparently strong batch effects and may need to be pre-processed individually. In the future, some of the latent structure in these datasets could be included in more sophisticated models based on MIRTH. For example, BrCa1 & BrCa2 samples had variable estrogen receptor (ER) positivity, a metabolically-relevant stratification that MIRTH does not account for.

### MIRTH finds latent information in existing metabolomics data

The ability of MIRTH to impute completely unmeasured metabolite features may reduce the cost and complexity of metabolomic profiling. As we have demonstrated, MIRTH can recover rank-normalized metabolite abundances which are of biological or clinical interest without requiring additional tissue or additional profiling. This enhances the potential for discovery by enriching publicly-available metabolomics data with additional metabolite features. A key consideration here will be sample size: our success in inferring positive-/negative-mode metabolites (Fig. 5) was in part related to the large number of samples available for training in this dataset.

More generally, the success of MIRTH implies that information on a restricted set of metabolites is sufficient for the imputation of a much larger set of metabolites. We envision the development of an assay which, instead of comprehensively profiling thousands of metabolites in a single sample, simply seeks to measure a small but highly informative subset of metabolites. With accurate measurements of a small panel of predictive metabolites combined with other datasets which measure a wider profile of metabolites, MIRTH or related methods in the future may be able to offer a much wider view of the metabolome at a greatly reduced cost. To that end, we have identified a small set of metabolites from which MIRTH can impute the ranks of many other metabolites, though further experimental work is required to determine the best metabolites for such an assay (Supplementary Information, Additional file 1: Table S7, Additional file 2: Fig. S9).

### MIRTH embeddings encode biological information

The decomposition of metabolomics data into a product of two low-dimensional matrices empirically captures some aspects of underlying biology. For example, the separation of tumor and normal samples in embedding space suggests that the MIRTH can learn general differences in the metabolome between these types of samples across cancer types. Similarly, since each embedding vector is a parts-based representation of the underlying data, the feature embedding vectors can be considered to represent different “components“ of the metabolome, which are then linearly combined according to the sample embeddings to recover the metabolite ranks of a given sample. Furthermore, MIRTH embeddings appear to discern chemical classification of metabolites without incorporating any additional information; for example, the separation of lipids and dipeptides in embedding space hints at high covariance between members of these metabolite classes. Future work could incorporate prior information into the matrix factorization, such as additional information on metabolite classes and structural similarities or on tissue and samples types.

The analysis of the embeddings also provokes questions about the general nature of correlations between metabolite pool sizes. While the existence and characteristics of such correlations are abundantly described in the literature [26–28], neither the mechanistic basis from which they arise nor their generality across biological contexts (e.g., different tissues, or different cancer types) is understood. The general principles which explain how metabolite pools co-vary have been difficult to discern because metabolite pools are subject to complex regulation, both by the metabolic enzymes that produce and consume them, as well as by more distal changes in metabolic flux or cellular physiology. The analysis of the MIRTH embeddings suggests that a relatively small set of linear combinations of metabolite pool sizes is sufficient to describe a large fraction of all the variation in the bulk metabolome. Understanding whether these embeddings are a reflection of a more fundamental, global relationship between metabolite pools is a worthy question for future investigation.

## Conclusions

The experiments described in this paper suggest that embedded within every metabolomics dataset is latent information about otherwise unmeasured metabolite features. Future work fully harnessing this latent information will likely require overcoming at least two challenges. The first relates to the inherently semiquantitative nature of metabolomics data, in which pool sizes are reported in ion counts that can only be compared across samples within the same feature. MIRTH overcomes this challenge by rank-transforming each metabolite feature within each batch. The cost of this solution is the loss of information on the magnitude of fluctuations in pool sizes. Future work which instead preserves relative magnitudes while remaining amenable to modeling across batches of data will prove powerful. MIRTH’s treatment of left-censored values could also be modified to draw on more sophisticated approaches that have been developed for within-dataset imputation. The second challenge relates to the very likely possibility that some correlations between metabolites will be specific to a particular tissue, disease, or other biological context. For example, certain metabolites may accumulate to a large extent in the context of mutations to genes coding for metabolic enzymes [29]. Further generalizations of NMF, including those which leverage additional information about the tissue source or disease of interest, prior information on the relationship of metabolites to one another in the metabolic network, or a secondary dataset (e.g., genomics, gene expression) may improve the predictive performance of MIRTH.

## Methods

MIRTH imputes missing metabolites across *K* metabolomics datasets. Each of $i = 1 \ldots K$ datasets $\varvec{D}_i$ contains the relative abundance levels of a subset $p_i$ of the total metabolites *P* measured in $n_i$ samples. The relative abundance levels are not comparable across metabolites or datasets. MIRTH overcomes this limitation by transforming relative abundance levels to a common scale (normalized ranks) within each batch. MIRTH then applies a nonnegative matrix factorization algorithm to the transformed matrix. By learning latent factors for each metabolite and sample, MIRTH is able to impute missing metabolites both within the same dataset and across datasets.

We have implemented MIRTH in Python v.$\ge$3.7. A script for MIRTH imputation, as well as a scaled-down demonstration of imputation performance, is available in our Github repository: https://github.com/reznik-lab/MIRTH. Experiments are run on Memorial Sloan Kettering Cancer Center’s High-Performance Computing Juno cluster. Figures are generated in R.

We assume that we are given *K* datasets, each representing one *batch* of data, i.e., a collection of samples from one metabolomics experiment. Each dataset records measurements of different sets of metabolites with different proportions of metabolite classes represented (Additional file 1: Fig. S5a). Every entry in the dataset contains a raw ion count for a specific metabolite detected in a sample. Ion counts below a threshold are not detected by the mass spectrometer. These counts are left-censored; the only information about them is that they are smaller than the smallest reported ion count in that dataset.

In the MIRTH method, the datasets individually undergo normalization and rank-transformation accounting for left-censoring as described below (Fig. 1a). Then, the preprocessed single datasets are aggregated into a multiple-dataset matrix, which is then factorized and imputed (Fig. 1b).

### Handling missing values in raw datasets

A targeted metabolomics dataset features two forms of missing data. The first class of missing data corresponds to metabolites which exist in a biological specimen at physiologically-relevant concentrations, but which have not been measured. We refer to these data as “missing metabolites,” emphasizing that their abundance is missing in all samples in a given dataset. The goal of MIRTH is to impute these missing data.

The second corresponds to metabolite measurements that are missing in some samples, but are measured in other samples in the same dataset. These missing values often represent instances where a metabolite’s abundance falls below the lowest quantified abundance of that metabolite across all samples. We refer to such instances as “left-censored” measurements. The extent of left-censoring varies by feature and by dataset (Additional file 2: Fig. S5b). MIRTH has specific procedures which handle left-censored data, as described below.

### Normalization

A variety of normalization techniques are used to control for variation in sample loading in metabolomics data [30]. We compared MIRTH’s imputation performance with total ion count (TIC) normalization, probabilistic quotient normalization (PQN) and without normalization enabled. MIRTH performs comparably with both normalization methods (Additional file 2: Fig. S5c). For all analyses described in the text, each dataset was preprocessed with TIC normalization. In TIC normalization, the ion count for every metabolite entry in sample *i* is normalized by

$$\begin{aligned} \varvec{D}_{\varvec{i}}^{\varvec{N}} = \frac{\varvec{D}_{i}}{f_{\varvec{D}_{\varvec{i}}}} \end{aligned}$$

where $\varvec{D_i^N}$ is the TIC-normalized sample vector, $\varvec{D_i}$ is the unnormalized sample vector, and $f_{\varvec{D_i}}$ is the TIC normalizer for sample *i*. The TIC normalizer is computed by summing the ion counts of all *j* metabolites in the sample,

$$\begin{aligned} f_{\varvec{D_i}} = \sum\limits_{j = 1}^{m} d_{i,j} + 0.5 \times \min (\varvec{D_i}) \times N_{{censored}_{\varvec{D_i}}} \end{aligned}$$

where $\min (\varvec{D_i})$ is the minimum value in dataset $\varvec{D_i}$ and $N_{censored}$ in the number of left-censored entries in the sample. Thus, left-censored values are included in the sum as one-half the the minimum value in the dataset.

### Rank-transformation

Since metabolomics only semi-quantitatively measures metabolite pool sizes, only one form of comparison (between two samples’ measurements of the same metabolite in the same dataset) is admissible. In contrast, comparisons of the abundance of two metabolites in the same sample or comparisons of the same metabolite across two samples from two different datasets are inadmissible. This is a fundamental limitation of mass spectrometry data, as a relative abundance measurement depends not only on a metabolite’s true concentration in the sample, but also on chemical and physical properties unique to the metabolite in question.

We rank the metabolite abundances of all the samples within each dataset. The samples with the highest ion count for a metabolite in the given dataset are ranked highest. The samples with the lowest ion count are ranked lowest. Left-censored values are tied for last rank. This distributes the sample abundances in each metabolite in the same way, allowing for the comparison of correlations between metabolite abundances in the same dataset. Rank-transformation also ensures that metabolite measurements can be compared across batches. Batch effects are typically assumed to take the form of shape and scale effects [31]. That is, given true values $Y^*$, the observed values *Y* are a latent linear function of the truth,

$$\begin{aligned} Y = f(\alpha Y^{*} + \beta ) \end{aligned}$$

where $f(\cdot )$ is either assumed to take some parametric form like a normal distribution or is treated nonparametrically and assumed only to be monotone without other specification. In either setting, the rank is invariant to batch effects: the monotonicity of $f(\cdot )$, whether it is normal or nonparametric, enforces that the ranks are preserved in expectation.

Since the ranks are invariant to the local batch effects, we only need to be concerned, statistically, with between-batch ranks. But here again, batch effects are by definition localized to a batch. Thus by converting measurements to within-batch ranks, the ranks should be comparable across batches, under typical batch effect assumptions.

The rank of the *i*th uncensored sample in a feature, $d_{i,j}$, is found by:

$$\begin{aligned} rank(d_{i,j}) = \frac{ 1 + \sum\limits_{i'=1}^{N_{total}} \mathbb {1}[d_{i,j} > d_{i',j}] }{1+N_{total}} \end{aligned}$$

where $N_{total}$ is the total number of samples in the feature. The rank for the left-censored entries in a feature is set to

$$\begin{aligned} \frac{0.5 \times (1 + N_{censored})}{1 + N_{total}} \end{aligned}$$

where $N_{censored}$ is the total number of censored samples in the feature. This rank is halfway between the minimum rank of uncensored samples and zero. This maps the features with only uncensored metabolites uniformly from 0 to 1. Following rank-transformation, features in each dataset have the same marginal distribution conditioned on having the same sample size. Rank-transformation results in higher performance compared to simply scaling each feature from 0 to 1 in each dataset when imputing features across datasets (Additional file 2: Fig. S5d).

### Nonnegative matrix factorization (NMF)

Nonnegative matrix factorization (NMF) is commonly used to obtain a low-rank approximation of nonnegative, high-dimensional data matrices [32]. NMF decomposes a matrix $\varvec{X} \in \mathbb {R}^{m \times n}, \varvec{X} > 0$ into $\varvec{W} \in \mathbb {R}^{m \times k}$ and $\varvec{H} \in \mathbb {R}^{k \times n}$ such that $\varvec{X} \approx \varvec{WH}, \varvec{W,H}>0$. When the rows of $\varvec{X}$ contain samples, then the columns of factor $\varvec{W}$ describe the relative contributions of each embedding vector to a sample [16] and can reveal clustering among samples [18]. Similarly when the columns of $\varvec{X}$ contain features (metabolites, in this case), the rows of factor matrix $\varvec{H}$ describes the relative contributions of the features to an embedding vector [16, 18].

To prepare the data for NMF, datasets preprocessed with normalization and rank-transformation are aggregated into a single aggregate data matrix $\varvec{X} \in \mathbb {R}^{m \times n}$, with rows corresponding to individual samples and columns corresponding to the complete set of metabolite measured across the batches. The sparseness of $\varvec{X}$ depends on the sparseness and feature overlap of the datasets that comprise it. For example, the matrix $\varvec{X}$ consisting of the nine metabolomics data datasets under consideration has 1727 samples, 1904 metabolite features, and 79.4% missing entries, including both missing metabolites and left-censored entries (Fig. 1c). In MIRTH, we formulate metabolite imputation of the unmeasured metabolites as a nonnegative matrix factorization problem which handles missing values,

1 $$\begin{aligned} \min _{\varvec{W,H}} \sum\limits_{i=1}^{m} \sum\limits_{j=1}^{n} (x_{ij} - w_{i}^{\top } h_{j})^2 \nonumber \\ \text {subject to }\mathbf {W, H} \ge 0 \end{aligned}$$

where the original data matrix is factored into the product of two low-dimensional matrices $W \in \mathbb {R}^{m \times k}$ and $H\in \mathbb {R}^{k \times n}$; missing $x_{ij}$ are omitted in the loss function. The structure of NMF naturally allows the imputation of missing values. Because $\varvec{W}$ and $\varvec{H}$ have fewer entries than $\varvec{X}$, not all the entries of $\varvec{X}$ are required to perform the decomposition. Provided the loss function is an entry-wise sum of losses, such as the least squares error used here, the matrix can be factorized by dropping the terms corresponding to the missing entries from the loss function [33]. This minimization problem is solved with SciPy’s optimize.minimize [34] and the autograd-minimize wrapper [35] with the L-BFGS-B algorithm [36]. Equivalently, a version of scikit-learn NMF that handles missing values can be used for faster runtimes [37]. Solving the optimization problem above produces two matrices $\varvec{W}$ and $\varvec{H}$ (Additional file 3), each with no missing entries, whose product $\varvec{\hat{X}} = \varvec{WH}$ also contains no missing entries. Following reconstruction, $\varvec{\hat{X}}$ is rank-transformed again to ensure feature measurements remain mapped uniformly between 0 and 1. These entries are the predicted metabolite ranks imputed by MIRTH.

### Cross-validation

In order to determine the optimal number of embedding dimensions *k*, we perform *v*-fold cross-validation; typically, $v=10$. We evaluate performance over a range of *k* in [1,80] or [1,60] in the single-dataset or across-dataset imputation cases, respectively.

For each *k*, we first identify the metabolite features that are available to use to score in dataset-wise cross validation, *i.e.* the metabolites in each dataset that are also measured in at least one other dataset. These available features are then equally partitioned into *v* folds (i.e., sets of metabolite features in each dataset on which we will test model performance with different sets of cross-validation parameters). Within each dataset, metabolites are randomly assigned to folds in order to reduce the amount of overlap between folds in different datasets (Additional file 2: Fig. S6a). Once the folds are defined, MIRTH loops through them, treating one at a time as unmeasured (masking all the fold’s features in the datasets where they appear), and factorize the resulting matrix. We then take the product $\varvec{WH}$ to recover $\hat{\varvec{X}}$, the imputed matrix with no missing data. Next, we compute the mean absolute error (MAE) between the true ranks of the metabolites in the fold (the metabolites we simulated as unmeasured) and the imputed ranks of those metabolites; the MAE is treated as the performance score for that fold. The scores for each fold are averaged, which yields a score for the particular value of *k*. This process is repeated for all *k*, and the value which results in the lowest MAE is chosen as the optimal number of embedding dimensions for the factorization (Additional file 2: Fig. S6b).

### Evaluating MIRTH’s performance

To evaluate MIRTH’s predictions of metabolite ranks, we mask a subset of metabolite measurements to simulate them as missing. After imputing the masked data with MIRTH, we compare the imputed metabolite ranks to the true ranks of the features that were simulated as missing. The chosen performance metric is Spearman’s rank correlation coefficient ($\rho$), computed between actual and predicted metabolite ranks. Correlation coefficients are computed separately for each metabolite that was simulated as missing. The resulting $\rho$ values are then summarized, either across all the metabolites simulated as missing in one experiment (to assess the overall prediction quality of a single imputation) or for the same metabolite across several repeated experiments (to assess metabolite-specific prediction quality). The $\rho$ values are Fisher z-transformed before summarization; the summarized z-scores are then inverse-z-transformed to yield summarized $\rho$ values. P-values are adjusted for multiple testing with Benjamini-Hochberg (BH) correction [38]. Details on how metabolite ranks are simulated as missing are included in their respective Results sections. Metabolites which are predicted with significant positive correlations with true ranks in more 90% of trials are deemed *well-predicted*. Metabolites are deemed *reproducibly* well-predicted if they are measured in at least four datasets and are well-predicted in at least three-quarters of the datasets in which they are measured.

## Supplementary Information

Additional file 1: Supplementary Tables S1-7. Compiled supplementary tables S1-7 with titles enclosed.

Additional file 2: Supplementary Figures S1-9. Compiled supplementary figures S1-9 with captions enclosed.

Additional file 3: Example MIRTH embedding matrices. Sample and feature embedding matrices (**W**, **H**) generated by applying MIRTH to 9-dataset aggregate with 30 embedding dimensions.

Additional file 4: Review history.

## Data Availability

All benchmarking datasets used in this study have been previously published [9, 21, 24]. MIRTH and a scaled-down demonstration of performance benchmarking are available on GitHub [39]: https://github.com/reznik-lab/MIRTH. Please see the licensing information in the repository for terms of use. The current MIRTH release is also deposited at a Zenodo repository [40]: 10.5281/zenodo.6803240.

## Appendix

### MIRTH experiments with simulated data

#### Generating Simulated data

Generating simulated data can accelerate the development of machine learning methods when acquiring real training data is costly [41]. The nine benchmark datasets in this study have variable size and sparsity. Evaluating MIRTH’s performance on realistic, synthetic datasets can help parse the effect of size and sparseness on performance under controlled conditions. As such, we simulated metabolomics datasets for benchmarking.

Our analysis confirmed that MIRTH’s multidimensional embedding space clusters related samples and metabolite features (Fig. 4a–c). Consequently, a simulated dataset $\{\varvec{s_1,..., s_N}\}$ would consist of *n* observations of a random *m*-dimensional Euclidean variable, such that the data belongs to *J* clusters.

We use a generative model to simulate metabolomics datasets, $\varvec{S} \in \mathbb {R}^{m \times n}$ where *m* is the number of samples and *n* is the number of metabolites. The dataset is generated as the product of simulated embeddings matrices, $\varvec{W_{sim}} \in \mathbb {R}^{m \times k}$ and $\varvec{H_{sim}} \in \mathbb {R}^{k \times n}$ where *k* is the number of embedding dimensions. To generate the embeddings matrices, a *k*-dimensional multivariate distribution of *J* cluster centroids is generated. This distribution is sampled to set the center of each embedding vector. The dataset is simulated as follows,

To generate $$\varvec{W_{sim}}$$:

2 $$\begin{aligned} c_{j_{clusters},k_{dims}}^{w}&{\mathop {\thicksim }\limits ^{i.i.d.}} Gamma(k = 100, \theta = 1) \nonumber \\ d_n&{\mathop {\thicksim }\limits ^{i.i.d.}} Cat(\varvec{\alpha })\\ w_{n,k_{dims}}&\thicksim Gamma(k = 100, \theta = 1) + c_{d_{n,}k_{dims}}^{w} \nonumber \end{aligned}$$

To generate $$\varvec{H_{sim}}$$:

3 $$\begin{aligned} c_{k_{dims},j_{clusters}}^{h}&{\mathop {\thicksim }\limits ^{i.i.d.}} Gamma(k = 100, \theta = .1) \nonumber \\ d_m&{\mathop {\thicksim }\limits ^{i.i.d.}} Cat(\varvec{\alpha }) \\ h_{k_{dims},m}&\thicksim Gamma(k = .1, \theta = 1000) + c_{k_{dims},d_{m}}^{h}\nonumber \end{aligned}$$

To generate $$\varvec{S}$$:

4 $$\begin{aligned} \varvec{S} = \varvec{W_{sim}} \times \varvec{H_{sim}} \end{aligned}$$

where $$\alpha _j =1, j= 1\ldots j_{clusters}$$.

The matrix product of these two embedding matrices constructs a simulated dataset. A set of feature names to sample from is defined. The columns of the dataset are randomly assigned a subset of these feature names. The rows of the dataset are given labels according to dataset and sample number. Once labeled, the dataset can then be combined into an aggregate data matrix ($\varvec{X}$) or used in a single-set imputation. We generated data with $j_{clusters}=5$ and $k =10$. The shape, *k*, and scale ($\theta$) of the sampled distributions for both the centroids and embedding matrix weights were chosen to create data similar to the 9 real datasets used for benchmarking.

#### Experiment 1: Size

In this experiment, nine simulated datasets of identical size to the real datasets were generated. To isolate the effect of dataset size on within-dataset imputation performance, First, we applied MIRTH to one dataset at a time, masking 10% of the features in half the samples (Fig. 2a). These masked entries in each dataset were imputed. Performance was better for larger datasets (Additional file 2: Fig. S7a), indicating that larger sample size improves within-set imputation performance. In general, the simulated data was imputed with much higher median $\rho$ than real data.

To isolate the effect of dataset size on *cross-set* imputation performance, we applied MIRTH to the aggregate dataset (9 merged simulated datasets), holding out entire features in one target dataset at a time (Fig. 3c). The cross-dataset imputation performance on the simulated data was roughly constant while the performance on the real data varied depending on the target dataset (Additional file 2: Fig. S7c). This simulation indicated that factors other than size contribute to performance of the across-set MIRTH imputation.

#### Experiment 2: Missingness

To test the effect of randomly-missing data on MIRTH’s imputation performance, identically-sized datasets were generated. Entries were randomly masked, with the proportion of masked entries ranging from 0 to 90% with a 10% step size. We evaluated single-set MIRTH imputation as usual (Fig. 2a). As the number of missing entries increases, the median performance ($\rho$) approached 0 (Additional file 2: Fig. S7b). The low performance stabilized around 50% missing entries.

We also constructed three datasets of identical size whose entries were randomly masked at different proportions (10%, 50%, 90%). We evaluated MIRTH’s across-set imputation performance as usual (Fig. 2c). As the number of randomly-missing entries increased, the median performance ($\rho$) decreased, approaching 0 (Additional file 2: Fig. S7d).

#### Experiment 3: Censoring

To simulate left-censoring, the ten lowest-valued entries in each feature in a dataset are masked. The imputed ranks of the simulated-censored measurements are compared to the true ranks of the same samples. Alternatively, the imputed ranks of the unmasked ten-lowest values can be compared to their true ranks.

We evaluated censoring imputation on real data (RC12). There are already many censored measurements in the dataset. Imputed ranks are consistently lower when their corresponding measurements are simulated as censored than when they are actually included in the training data (Additional file 2: Fig. S7e). Since some features have only one or a few measurements, a cluster of true ranks appears near 0.5.

This evaluation scheme was also run on a simulated dataset with 30% of entries randomly missing. Similarly, imputed ranks are lower when the data point is simulated censored than when the data point is actually included in the training data (Additional file 2: Fig. S7f). These findings confirm that MIRTH preserves the low ranks of censored data, which ultimately correspond to low metabolite abundance.

### Using MIRTH to identify a set of core predictive features

Given the success of MIRTH for within- and across-dataset imputation, we aim to identify a small set of metabolites that could inform the predictions of a larger set of metabolite ranks. With further development, precise measurements of this small set of metabolites could constitute an assay that reduces the cost of profiling large parts of the metabolome.

One approach is to find the set of features that maximizes the mutual information of the core features and batch features. In other words, we want to find the set of training features which reduces the uncertainty about all the features in the dataset. The mutual information, $\varvec{I}$, between sets *X* and *Y* is defined as

5 $$\begin{aligned} \varvec{I(X,Y)} := \varvec{H(X) - H(X|Y)} \end{aligned}$$

where $\varvec{H}$ is the entropy of the set [42].

Let $\varvec{C}$ be the set of core features selected for a dataset, and let $\varvec{F}$ be the set of all features measured in that dataset. To find the best set of core features, we want to find

6 $$\begin{aligned} \displaystyle {\max _{\varvec{C}} \varvec{I(C,F)} } \end{aligned}$$

Choosing a set of features that are maximally informative through mutual information is hard to estimate statistically [43]. However, we can approximate the concept by instead minimizing the predictive error between the true data and the data predicted by MIRTH trained under the core features. We want to find the set that minimizes the mean absolute error (MAE),

7 $$\begin{aligned} \displaystyle {\min _{\varvec{C}} MAE(\hat{\varvec{X}}_{test},\varvec{X}_{test})} = \displaystyle {\min _{\varvec{C}} \frac{1}{n}{\sum _{i=1}^{n} |\hat{x}_{i} - x_{i}|}} \end{aligned}$$

with $\hat{\varvec{X}}_{test}$ the predicted ranks of the testing data trained on the set of core features $\varvec{C}$, and $\varvec{X}_{test}$ the true ranks of the testing data.

As we augment the set of core features, we choose $f_{i}$ such that

8 $$\begin{aligned} \displaystyle {\min _{\varvec{{f_i}}} MAE(\hat{\varvec{X}}_{test},\varvec{X}_{test})} \end{aligned}$$

where $\hat{\varvec{X}}_{test}$ is imputed by the model trained on $\varvec{C} \cup f_{i}$.

To approximate the solution to this minimization problem, we use a greedy forward step-wise selection algorithm (Algorithm 1). In doing so, we assume that the problem is submodular, *i.e.* that the marginal gain in performance from adding an extra element *c* to the set of core features $\varvec{C}$ is greater than the marginal gain in performance when *c* is added to any superset of $\varvec{C}$ [44]. Submodularity is a reasonable assumption since choosing the set of core features through mutual information is submodular [45].

Before selecting core features, we calculate the by-dataset baseline MAE values of MIRTH imputation with no training features in the target dataset (due to non-uniformity cause by left-censoring, the MAE values of rank-transformed are not necessarily 0.25). Selection of a core feature begins with a list of candidate features, consisting of all features in the aggregate dataset except for the current core features (when selecting the first core feature, this set is empty). Each candidate feature, along with any previous core features, are used as training data for each target dataset. All other features are held out from the target dataset. MIRTH is applied to the remaining data (including all other non-target datasets), and MAE is computed. If a candidate feature is not present in a target dataset, the previous baseline MAE is substituted. Once all candidate features have been assessed in all datasets, the average MAE across for each candidate is computed across datasets. The candidate feature yielding the lowest average MAE is selected as the next core feature; the by-batch MAE values become the new baseline MAE values. This process is repeated for the desired number of candidate features.

We identified 50 core metabolite features using Algorithm 1. As expected, the average MAE decreased as the size of the set of core features increased (Additional file 2: Fig. S9). The selection algorithm is biased to first pick features that are measured in many datasets, before quickly becoming biased toward features measured in only one dataset (Additional file 2: Fig. S9). The first of the 50 metabolites, phosphoethanolamine, is involved in phospholipid metabolism. The core features span various metabolite classes: they consist of 12 lipids, 7 xenobiotics (5 of which are drugs), 6 peptides, 4 amino acids, 3 cofactors and vitamins, 1 carbohydrate, 1 nucleotide, and 16 uncharacterized metabolites (Additional file 1: Table S7).

Algorithm 1’s tendency toward choosing metabolites that are measured in few datasets somewhat hinders the utility of the list of 50 metabolites it has nominated. It may also suggest the existence of cancer-type-specific core metabolite features. Nonetheless, the selected set serves as a template for future investigation. Further refinement of Algorithm 1, as well as in-vitro experiments to determine the reliability of measuring these metabolites, could help curate or expand the set of core metabolite features.
