# Supplementary material for: MIRTH: Metabolite Imputation via Rank-Transformation and Harmonization
Source: Genome Biol. 2022 Sep 1;23:184. doi: 10.1186/s13059-022-02738-3 (PMC9438248; doi:10.1186/s13059-022-02738-3)
Supplement: Supplementary file 2 — Additional file 2: Supplementary Figures S1-9. Compiled supplementary figures S1-9 with captions enclosed. [file 13059_2022_2738_MOESM2_ESM.pdf]

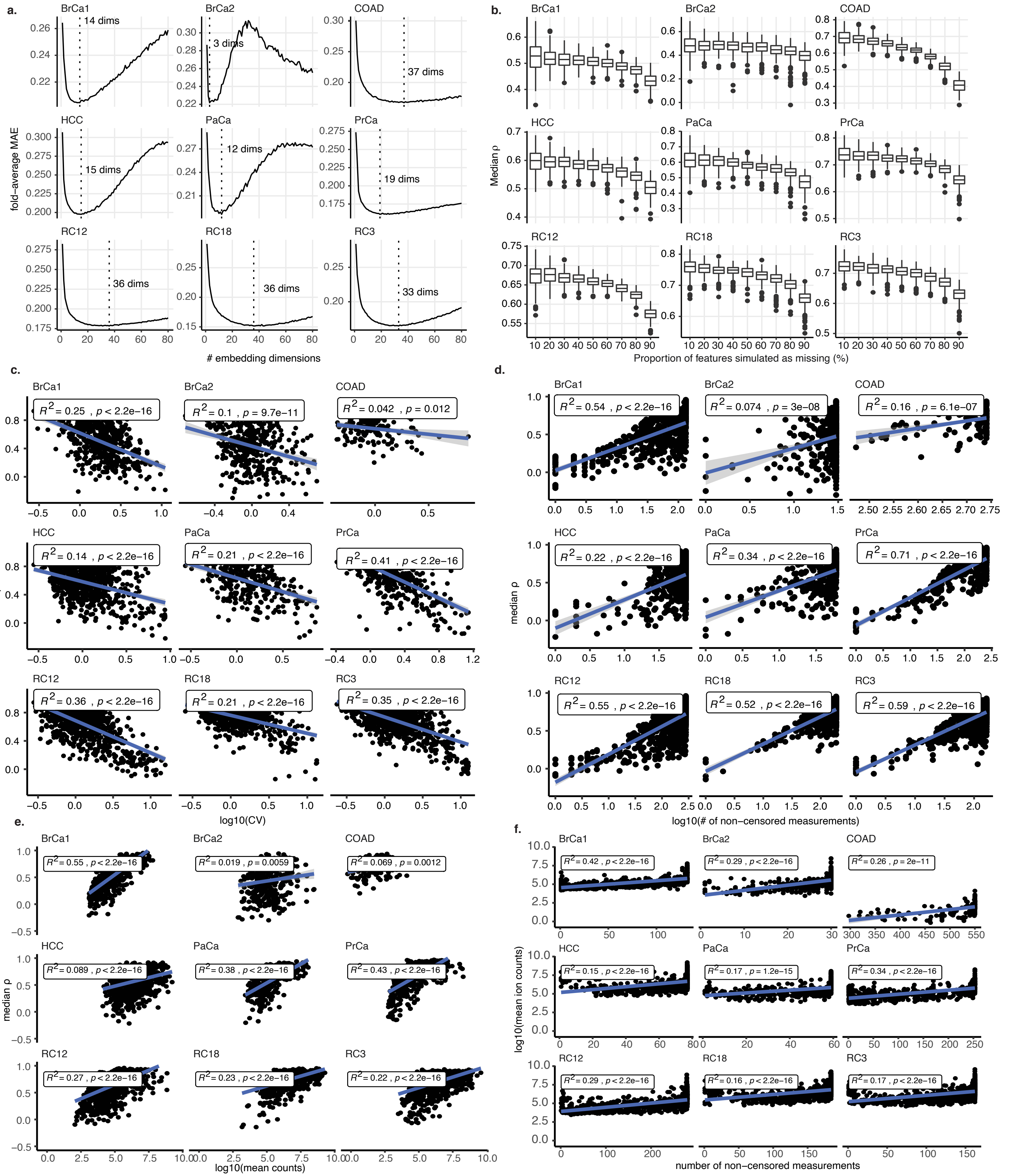

Figure S1: Supplemental Figure 1. **a.** Mean absolute error, averaged across cross-validation folds, as a function of number of embedding dimensions in single-set *in-silico* imputation experiments. The error-minimizing number of embedding dimensions varies by dataset, partially depending on dataset size. **b.** Imputation performance decreases as a function of the proportion of features simulated missing in a single-set imputation. **c.** Median by-metabolite performance as a function of raw data coefficient of variation (CV, log-scaled) in single-set imputation. **d.** Median by-metabolite performance as a function of the number of non-censored measurements in the dataset (log-scaled). **e.** Median by-metabolite performance as a function of mean raw ion counts (*i.e.* relative abundance). Metabolites with lower count values are biased for worse performance, though likely still due to a larger extent of censoring, as (**f.**) the two are correlated. Extent of censoring decreases along the x-axis.

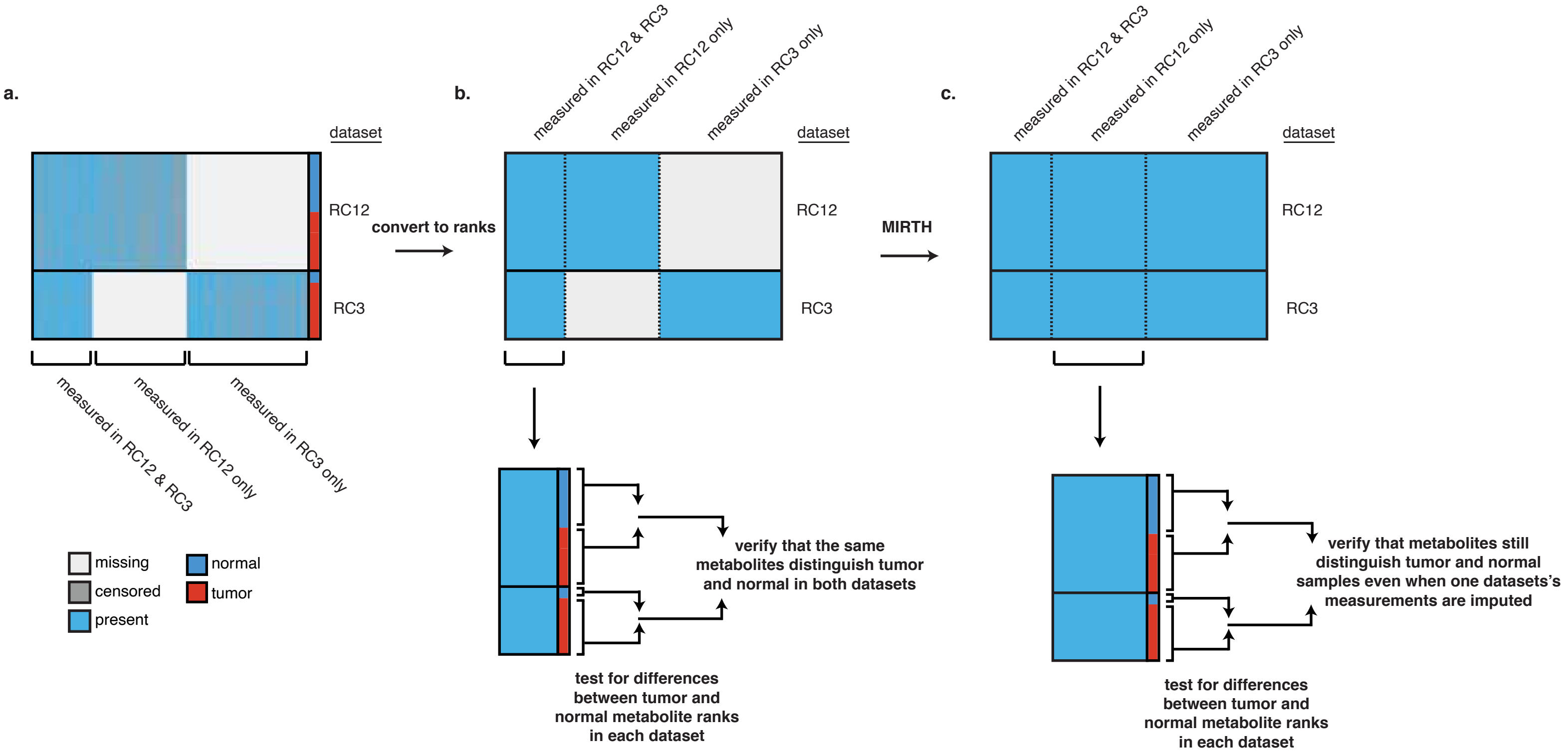

Figure S2: Supplemental Figure 2: Procedure for determining whether imputed metabolite measurements can still distinguish tumor and normal samples. **a.** Data is preprocessed and aggregated as usual. **b.** Metabolites that are differentially abundant between tumor and normal samples in each dataset are determined and compared to ensure the similarilty of the datasets. **c.** The differential abundance analysis is repeated with metabolites that were entirely unmeasured but imputed with MIRTH in one of the datasets to ensure that imputed ranks align with underlying biological characteristics of the data.

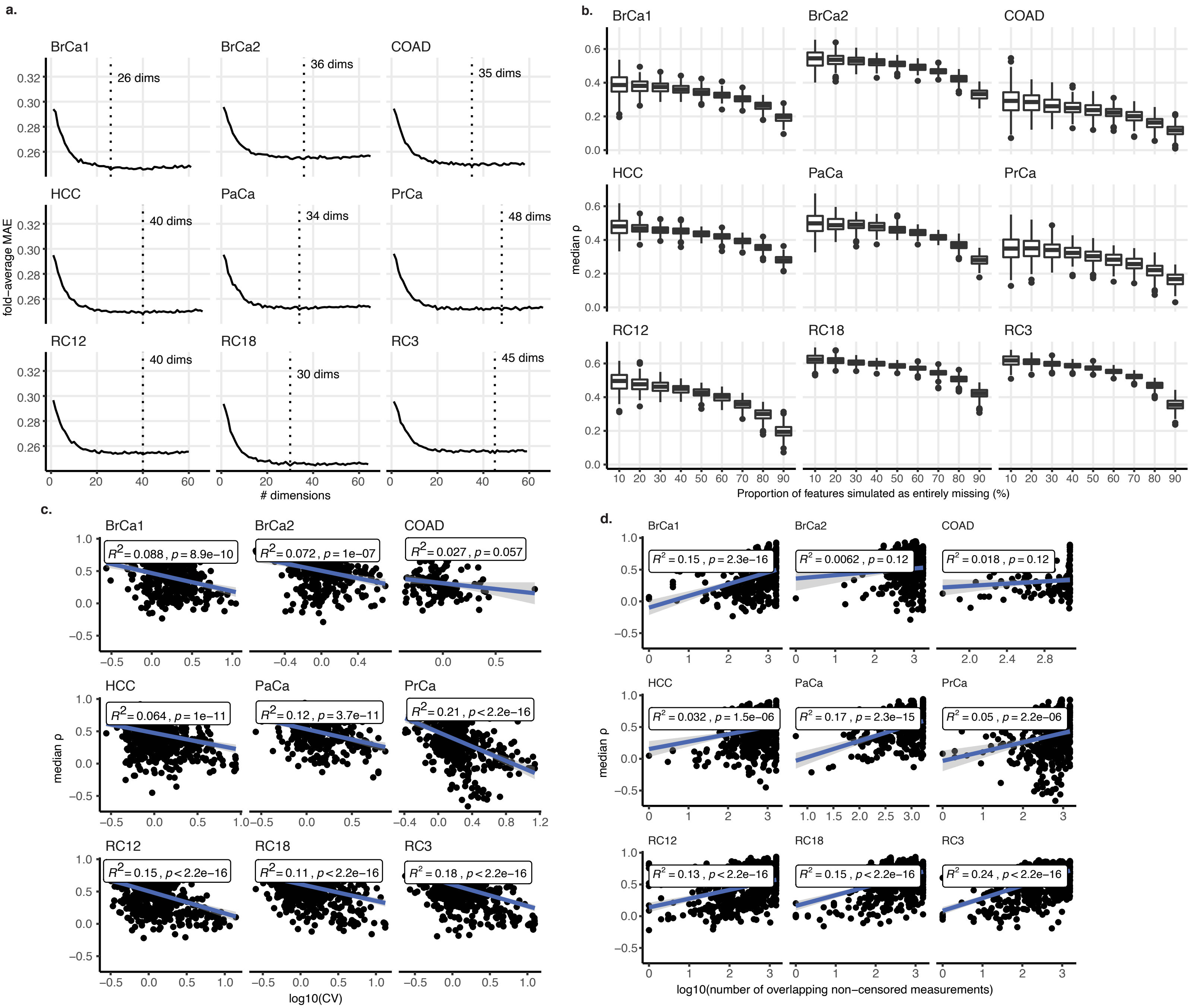

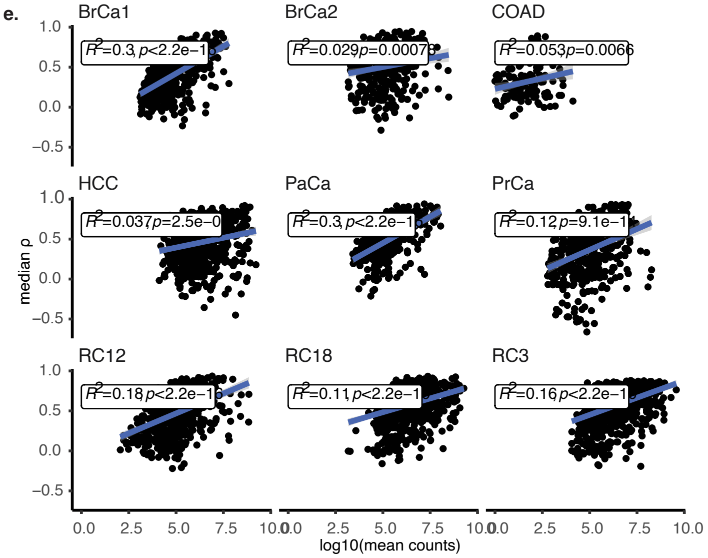

Figure S3: Supplemental Figure 3. **a.** MAE as a function of number of embedding dimensions in across-dataset *in-silico* imputation experiments. The error-minimizing number of embedding dimensions varies by target dataset, but 25-30 embedding dimensions are likely to yield equivalent-to-optimal imputation performance. MAE increases slowly due to the large size of the aggregate dataset. **b.** Median performance by target dataset worsens as a function of the proportion of features simulated as missing in the target. **c.** CV of predicted features may explain performance in some target datasets. **d.** The extent of overlap between features in the target dataset with uncensored measurements in other datasets may explain variation in performance. **e.** Metabolites with low raw ion counts are biased toward worse performance, though likely due to a larger extent of censoring for those metabolites (Figure S1f).

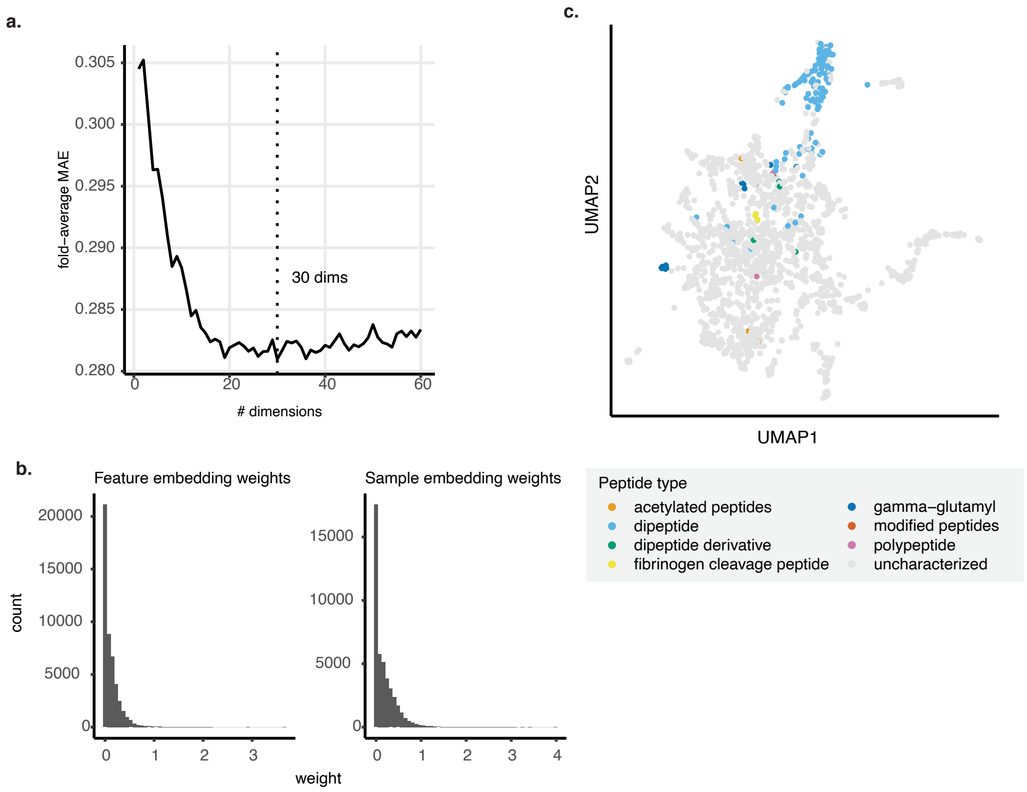

Figure S4: Supplemental Figure 4. **a.** MAE vs. number of embedding dimensions for a whole-dataset imputation. **b.** UMAP of feature embedding matrix annotated to show that clustered peptides are dipeptides. **c.** Histograms of weights in sample and feature embedding matrices. Few samples and features have weight > 1.

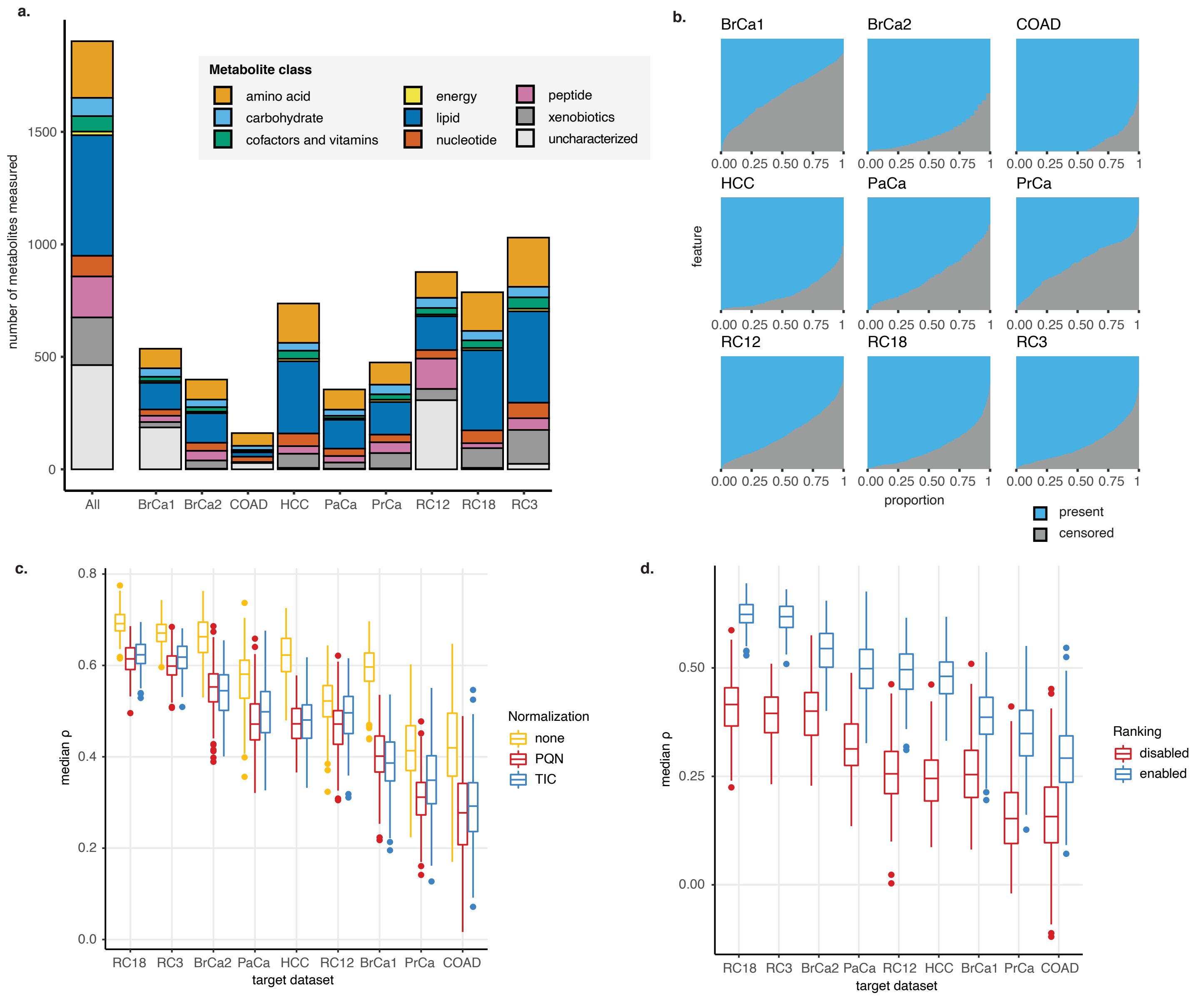

Figure S5: Supplemental Figure 5. **a.** Breakdown of metabolite classes measured in each dataset. **b.** Each dataset contains censored features. **c.** Probabilistic quotient normalization (PQN) and total ion count (TIC) normalization result in roughly equivalent performance; using no normalization results in higher performance but does not control for sample loading. **d.** Rank-transformation, compared here to scaling all normalized ion counts to a range of (0,1], is crucial to across-dataset imputation performance.

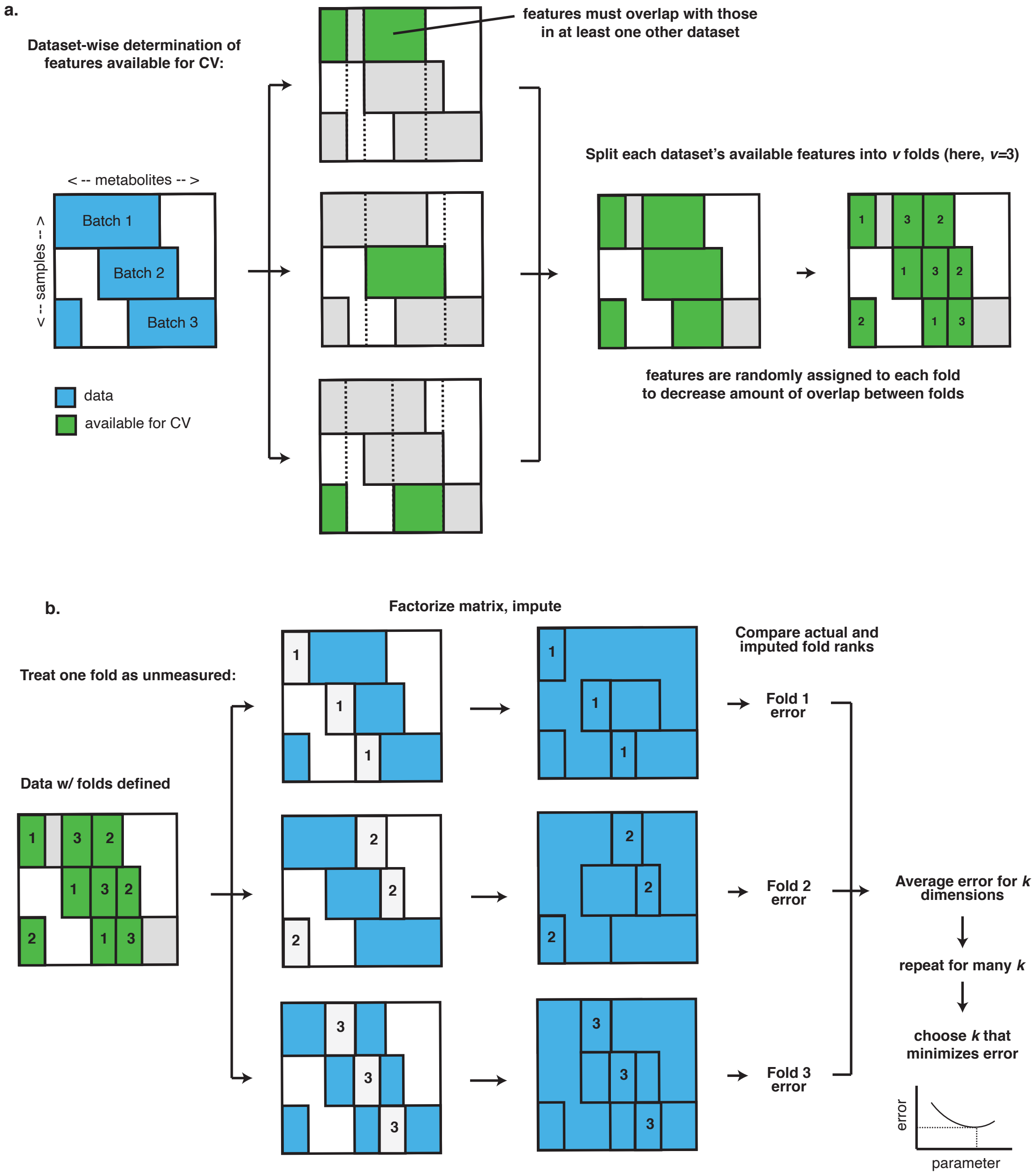

Figure S6: Supplemental Figure 6. **a.** Process of defining folds for cross-validation. The features available for cross-validation are determined in each dataset on the basis of overlap with at least one other dataset. Then, features are assigned to *v* folds randomly in order to reduce the number of features that overlap between folds. **b.** Schematic of cross-validation scoring procedure. For each fold, the measurements in the fold are masked (treated as unmeasured) and the remaining data is imputed. The error between the imputed measurements and the true measurements is computed. The errors are then averaged across folds, which yields an error for the number of embedding dimensions employed. The process is repeated for multiple numbers of embedding dimensions. The number of dimensions that minimizes the error is chosen as the optimal one to be used in the imputation of the data.

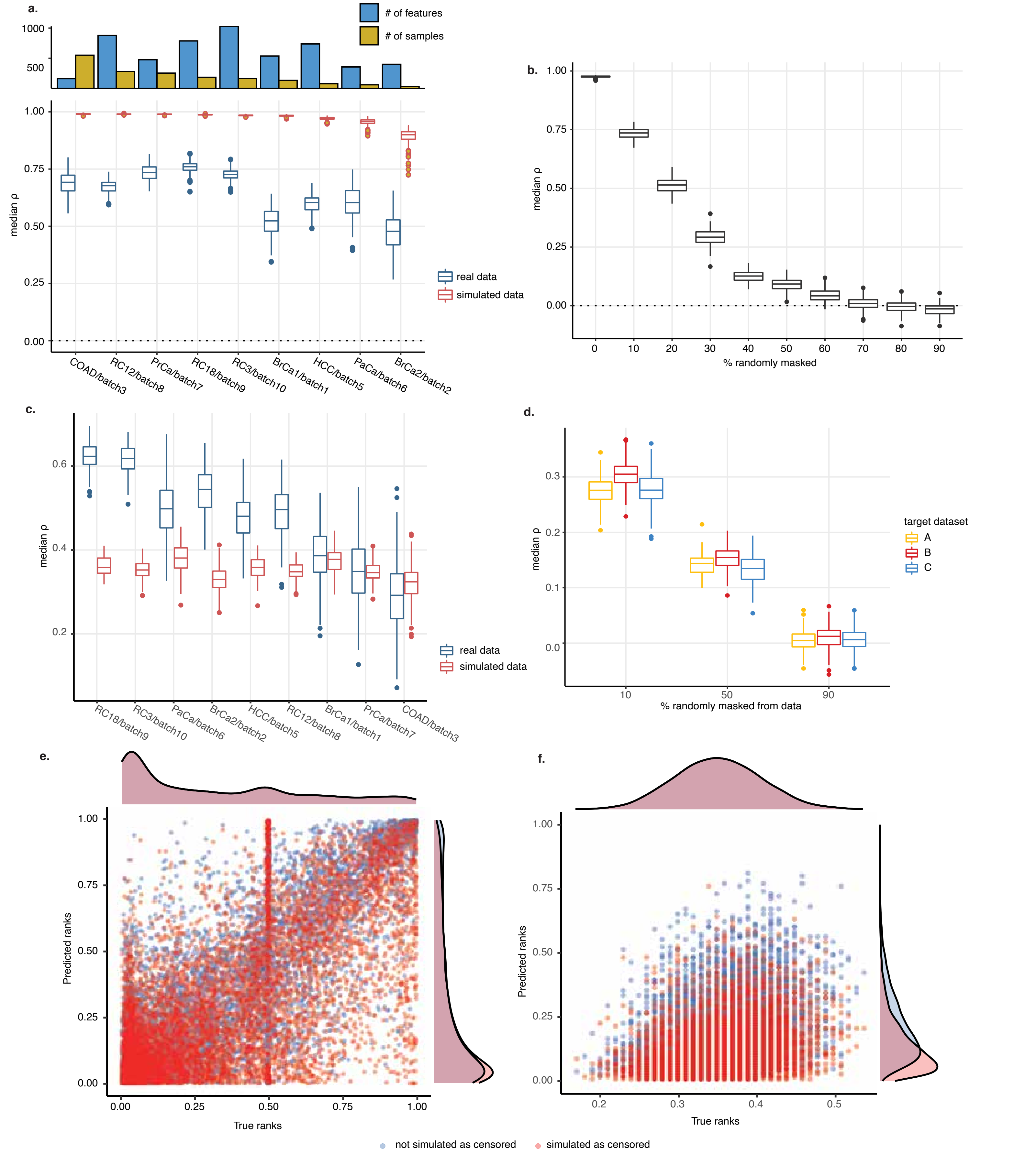

Figure S7: Supplemental Figure 7. **a.** Single-dataset imputation of size-matched simulated data, with the performance of the simulated data and of the corresponding real data both shown. **b.** Median performance on single-set imputation of identically-sized datasets as a function of percentage of data randomly masked. **c.** Median performance of across-dataset imputation of size-matched simulated data compared to real data. **d.** Median imputation performance in across-dataset imputation of simulated data as a function of the percentage of data randomly masked. **e.** True ranks versus imputed ranks of ten smallest measurements in each feature of RC12 when they are simulated as censored (masked) and when they are not masked from the data before MIRTH imputation (not simulated as censored). **f.** True ranks versus imputed ranks of ten smallest measurements in each feature of a simulated dataset with 30 percent missing entries when they are simulated as censored and when they are not masked from the data before MIRTH imputation.

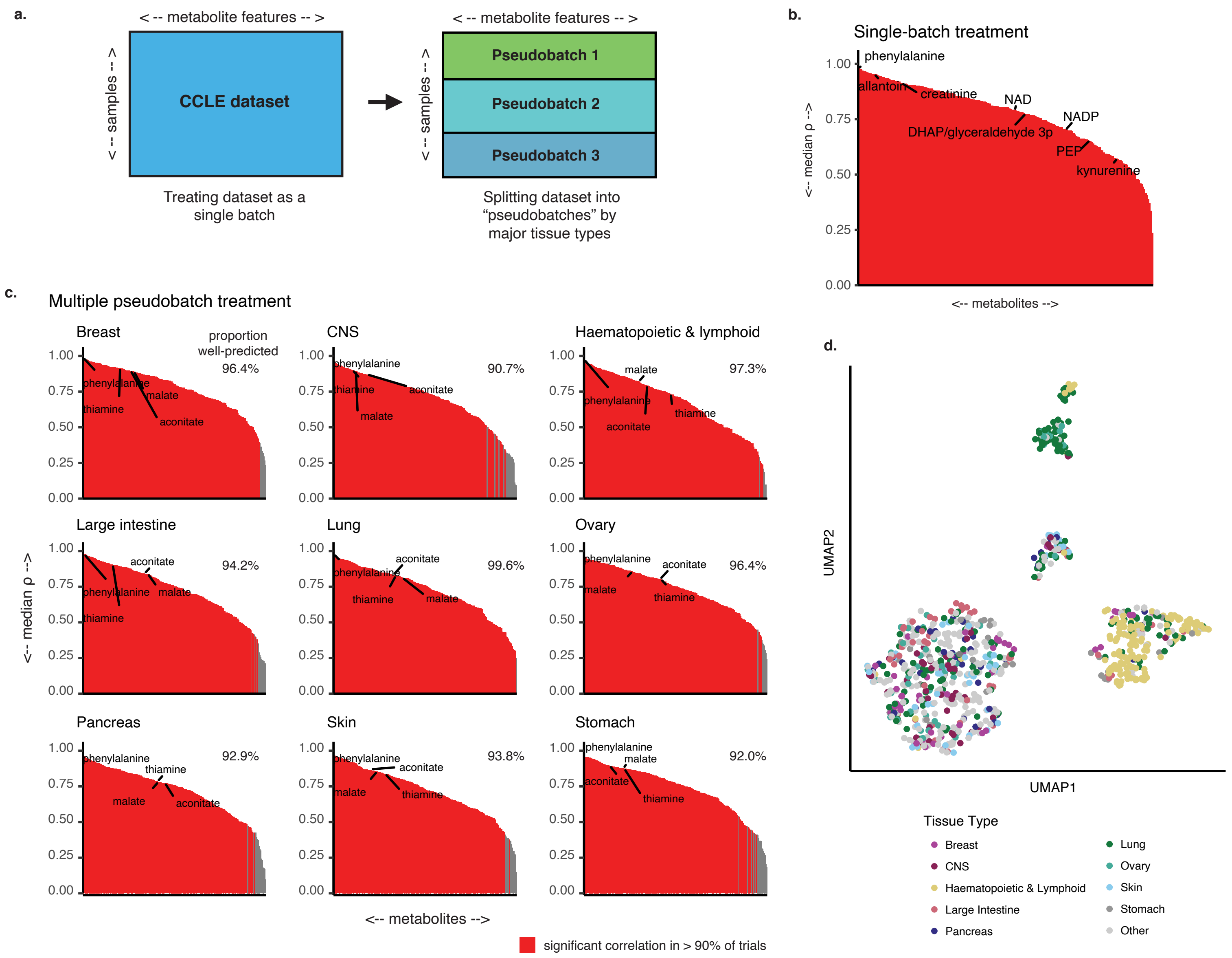

Figure S8: Supplemental Figure 8. **a.** The CCLE dataset can be treated as a single batch, or split into multiple "pseudobatches" delineated by major tissue type. Only pseudobatches with more than 30 samples are included. **b.** MIRTH predicts all metabolites in the CCLE dataset when it is treated as a single batch. Performance was evaluated as in Figure 2. **c.** MIRTH accurately predicts metabolites across CCLE pseudobatches well, with 90.7-99.6% of metabolites in each target batch being well-predicted. Performance was evaluated as in Figure 3. Percentage of well-predicted features in each target pseudobatch is shown. **d.** MIRTH may separate some samples in the CCLE dataset by tissue type, with some separation of haematopoietic & lymphoid tissue and lung tissue. Separation between other tissue types is less discernible.

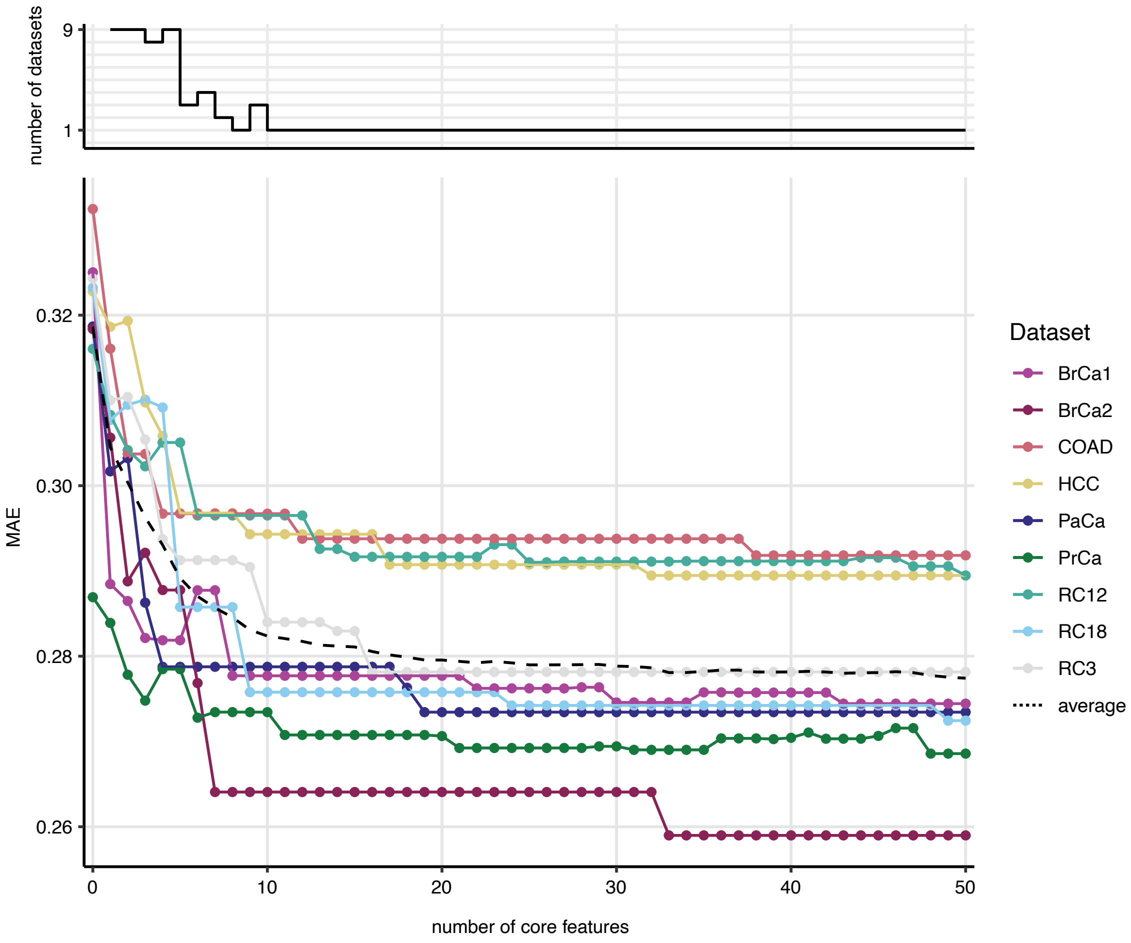

Figure S9: Supplemental Figure 9. Dataset-wise MAE when training on core features. MAE decreases on average as predictive features are added to training set. The new chosen features are measured in fewer datasets as the set of core features grows.
