# Supplementary material for: MIRTH: Metabolite Imputation via Rank-Transformation and Harmonization
Source: Genome Biol. 2022 Sep 1;23:184. doi: 10.1186/s13059-022-02738-3 (PMC9438248; doi:10.1186/s13059-022-02738-3)
Supplement: Supplementary file 4 — Additional file 4: Review history. [file 13059_2022_2738_MOESM4_ESM.docx]

Review History

**First round of review**

**Reviewer 1**

**Were you able to assess all statistics in the manuscript, including the appropriateness of statistical tests used?**

All statistical tests and methods formulations looked correct to me.

**Were you able to directly test the methods?**

No.

**Comments to author:**

Freeman et al. presents a rank-based imputation approach, MIRTH, to address the problem of missing measurements across metabolomics experiments. Particularly, MIRTH using a latent representation of the metabolomics imputes unmeasured metabolites across samples in different experiments, even for completely absent metabolites in a particular dataset, providing a data-driven way to fill the gaps across datasets.

MIRTH contrasts with existing approaches which mostly focus on the prediction of metabolites within a sample, this therefore presents many potential important applications in many contexts, such as cancer metabolomics. The approach is interesting and well fundamented. The work is performed to an excellent standard, well written and presented.

I have very few comments, where my main concern — which is general to any imputation approach — is how accurately can MIRTH predict metabolites that show specific context-dependent variation? For example, if a metabolite accumulates abnormally due to specific mutations in certain cancers, are the latent spaces learnt by MIRTH enough to provide this resolution of predictions?

Main comments:

- From what I could gather the metabolomics datasets were assembled from PMID:29396322, where the following is stated: "(...) we imputed any missing measurements for a compound with the minimum measured value of that compound". Thus, have the authors confirmed if metabolites missing values have a potential impact in the predictions of MIRTH? Since missing values were imputed this represents a potential leakage for the cross-validation approaches. I think it would be at least important to assess the level of missingness of metabolites across datasets.

- I found the following observation: "We observed that metabolite features with larger coefficients of variation (in the raw, non-rank-transformed data) tended to have lower imputation accuracy" interesting and potentially related to my main concern stated before. Because, for metabolites with very specific context dependent variability, which might be exactly those with highest variability, MIRTH may be underpowered to predict their abundance, even in large scale datasets.

- Have the authors seen any bias in prediction related with relative abundance of the metabolites, i.e. if more abundant metabolites are better predicted?

- The analysis across different cancers is interesting. Nevertheless, since each experiment seems to cover a different cancer/tissue type, a joint analysis of all datasets can discern biological variation from technical batch effects, such as those likely observed in PrCa dataset. Therefore, it would be important to have an application of MIRTH to a pancancer metabolomics dataset acquired using a similar platform, such as potential study could be the cancer cell line metabolomics dataset (PMID:31068703).

Minor comments:

- "Variation in MIRTH performance across datasets was partly explained by dataset size; imputation performance was better for larger datasets (Figure 2c)". What is shown in Figure 2c does not seem to support this statement clearly. Could the authors elaborate and comment on other potential technical factors, such as experimental type and protocols used, that could explain this difference?

- Could the authors explain why the fold-avg MAE worsens so quickly in Figure S1a in contrast to Figure S3a?

**Reviewer 2**

**Were you able to assess all statistics in the manuscript, including the appropriateness of statistical tests used?**

No.

**Were you able to directly test the methods?**

No.

**Comments to author:**

Overall this paper addresses an important need in the field of metabolic profiling: cross dataset comparison and imputation of missing data. This work is likely to advance the field substantially. The methods seem adequate, even though not completely novel (see below):

The proposed methodology is comprised of the following pillars:

- Samples are normalized within each dataset first with a sample specific normalization factor that takes into account the proportion of missing values. Then each metabolite is converted to ranks across samples (within a dataset), with a minimum rank determined for missing values.

- Then the datasets are combined together; there are metabolites reported in one dataset but not in another and these are imputed. Non-negative matrix factorization is used with a MSE error loss function where missing variables are dropped. This gives a low dimensional embedding for all metabolites including the unmeasured ones - which can now be estimated.

In further experiments the Authors hold out various proportions of metabolites from datasets and then compare the predicted rank to the true rank. The optimal embedding size is estimates from cross validation (typically dim=30). Results show that imputed metabolites maintain normal vs tumor differential expression ranking with another dataset with known data. Finally a leave-one-dataset-out cross study validation is performed (figure 3).

Some concerns should be addressed by the Authors:

A google search reveals this paper: https://www.ncbi.nlm.nih.gov/pmc/articles/PMC8510447 "NMF-Based Approach for Missing Values Imputation of Mass Spectrometry Metabolomics Data". From a cursory look, the approach appears to be similar, apart from the ranking method applied prior to NMF. A comparison is needed to differentiate this method from the proposed one and possibly a performance comparison should be entertained.

Some of the resulting figures are not very clearly explained. For example, in figure 2c are the metabolites (or metabolite x dataset trial combinations?) ranked on the x-axis by proportion of missing samples? The caption says "as number of samples increased along the x-axis the proportion of well-predicted metabolites in a batch increased as well". This requires clarification. Also in the predicted vs true rank comparisons (e.g. figure 2f), it is not clear what each point actually represents? If each point is a trial run with different proportions of metabolites simulated as missing, this should be clearly indicated.

Page 10, section 4.3: "comparisons of the abundance of two metabolites in the same sample... are inadmissible". I assume this is without appropriate pre-processing but additional explanation is again required. Also, it is briefly mentioned in section 2.3 that batch effects within each dataset can affect the results. Stating that PCa dataset revealed 3 batches is fine but the method doesn't seem to account for this. Obviously, this is important as it will affect the ranks significantly.

Some of the linear combinations derived to predict some key metabolites should be shown. Are the linear combinations obtained from the same metabolites in different datasets that are widely different? Are there key metabolites that are highly important for predicting many of the missing ones? i.e. Some biological interpretation of the results would be of great relevance.

**Authors Response**

**Point-by-point responses to the reviewers’ comments:**

Reviewer #1:

Freeman et al. presents a rank-based imputation approach, MIRTH, to address the problem of missing measurements across metabolomics experiments. Particularly, MIRTH using a latent representation of the metabolomics imputes unmeasured metabolites across samples in different experiments, even for completely absent metabolites in a particular dataset, providing a data-driven way to fill the gaps across datasets.

MIRTH contrasts with existing approaches which mostly focus on the prediction of metabolites within a sample, this therefore presents many potential important applications in many contexts, such as cancer metabolomics. The approach is interesting and well fundamented. The work is performed to an excellent standard, well written and presented.

I have very few comments, where my main concern — which is general to any imputation approach — is how accurately can MIRTH predict metabolites that show specific context-dependent variation? For example, if a metabolite accumulates abnormally due to specific mutations in certain cancers, are the latent spaces learnt by MIRTH enough to provide this resolution of predictions?

Main comments:

- From what I could gather the metabolomics datasets were assembled from PMID:29396322, where the following is stated: "(...) we imputed any missing measurements for a compound with the minimum measured value of that compound". Thus, have the authors confirmed if metabolites missing values have a potential impact in the predictions of MIRTH? Since missing values were imputed this represents a potential leakage for the cross-validation approaches. I think it would be at least important to assess the level of missingness of metabolites across datasets.

*Reply: Thank you for the positive commentary.*

*In the manuscript cited by the referee (Reznik et al., Cell Systems 2018), the raw data (ion counts) were indeed subject to processing, including imputation to the minimum measured value. However, in this work, we reverted back to the original raw ion counts, which include information on which measurements were left-censored. This is clarified on Page 3 in the Results.*

*With regard to the question of whether specific metabolites accumulate to a large extent in the context of specific genetic mutations, we do not currently have the ability to interrogate this question. Nearly none of the samples used in this study have associated genomic data available to us, and we are not aware of any samples with high-effect-size mutations such as loss of FH/SDH or mutation of IDH1/2. We have mentioned in the Discussion on Page 9 that this is an intuitive and valuable direction for future, multimodal analyses of metabolism.*

- I found the following observation: "We observed that metabolite features with larger coefficients of variation (in the raw, non-rank-transformed data) tended to have lower imputation accuracy" interesting and potentially related to my main concern stated before. Because, for metabolites with very specific context dependent variability, which might be exactly those with highest variability, MIRTH may be underpowered to predict their abundance, even in large scale datasets.

*Reply: Though it's true that high coefficient of variation or large extent of censoring seem to worsen predictions for certain features, we have also demonstrated that MIRTH quantitatively recapitulates variability owing to biological differences, e.g. differential abundance of key metabolites between tumor and normal samples (Figures 3a, b). The effect sizes of tumor vs. normal differences in metabolite levels are typically quite large and can exceed the variability across only tumor (or across only normal) tissues, indicating that MIRTH can capture large-magnitude variability in metabolite levels. This demonstration, we believe, adds confidence to MIRTH predictions for metabolites whose level depends on biological context, provided that there are enough training samples across a diversity of biological contexts. We have emphasized this point on Page 8 of the Discussion.*

- Have the authors seen any bias in prediction related with relative abundance of the metabolites, i.e. if more abundant metabolites are better predicted?

*Reply: Thank you for the question. In some datasets, metabolites with a higher mean raw ion count (i.e., relative abundance) tend to be better-predicted in within-set and across-set imputation (Figure R1a, b below). However, we also note that features with lower mean raw ion counts tend to experience more censoring (Figure R1c below). In other words, there is an inverse correlation between a feature’s extent of censoring and its mean raw ion count, as features with lower count values are more likely to have measurements that fall below the MS instrument’s detection threshold. We therefore believe that the observed bias in prediction related to relative abundance more likely reflects the imputation performance’s dependence on the extent of censoring, especially since all relative abundance measurements are rank-transformed before imputation. We have added the panels of Figure R1 to Figures S1 and S3.*

- The analysis across different cancers is interesting. Nevertheless, since each experiment seems to cover a different cancer/tissue type, a joint analysis of all datasets can discern biological variation from technical batch effects, such as those likely observed in PrCa dataset. Therefore, it would be important to have an application of MIRTH to a pancancer metabolomics dataset acquired using a similar platform, such as potential study could be the cancer cell line metabolomics dataset (PMID:31068703).

*Reply: Thank you for the suggestion. We have completed a series of benchmarks using the CCLE pan-cancer metabolomics dataset (Li et al., Nature Medicine 2019). We demonstrated that MIRTH robustly imputes missing metabolite measurements when treating the dataset as one batch (Figure R2b). To simulate across-dataset imputation, we also split the CCLE dataset into 9 “pseudobatches,” delineated by the major tissue types (Figure R2a). Using these pseudobatches, we benchmark MIRTH as we would for other across-dataset imputation tasks (Figure 3). MIRTH accurately imputes 90.7-99.6% of metabolites in each pseudobatch, again demonstrating its effectiveness at learning metabolite pool size correlations across tissue types (Figure R2c). Finally, we applied MIRTH to the whole CCLE dataset to generate low-dimensional sample and feature embeddings. We note that, as with the MSKCC datasets, there is variable separation of tissue types, with haematopoietic/lymphoid and lung tissue samples clustering but other separation being less discernible.*

*We have added the above figure to the Supplementary Information as Figure S8 and describe it in the text on Page 6 of the Results.*

Minor comments:

- "Variation in MIRTH performance across datasets was partly explained by dataset size; imputation performance was better for larger datasets (Figure 2c)". What is shown in Figure 2c does not seem to support this statement clearly. Could the authors elaborate and comment on other potential technical factors, such as experimental type and protocols used, that could explain this difference?

*Reply: Thank you for pointing this out. Although dataset size doesn’t fully explain the variation in median performance, Figure 2c does show that MIRTH typically performed better on the five datasets with the most samples compared to the four with fewer samples. We have clarified this wording on Page 4 of the Results.*

*As you point out, there may well be technical factors that explain the variation in performance. The best-performing dataset, COAD, was the only dataset which was not profiled using the Metabolon platform; it also contains relatively few features, including almost no lipids. As we note in the Discussion, samples in the BrCa1 and BrCa2 datasets, both relatively poorly-performing, had variable estrogen receptor (ER) positivity, a metabolically-relevant stratification which MIRTH does not account for. We have further outlined these differences on Page 8 of the Discussion.*

- Could the authors explain why the fold-avg MAE worsens so quickly in Figure S1a in contrast to Figure S3a?

*Reply: Figures S1a and S3a both detail the fold-average MAE as a function of the number of embedding dimensions sampled in v-fold cross-validation. Figure S1a is concerned with single-batch (i.e. within cancer-type/dataset) imputation, whereas Figure S3a is concerned with across-batch (i.e. across dataset/cancer type) imputation using all 9 datasets in aggregate. Single batches are much smaller than the 9-dataset aggregate - both in numbers of samples and numbers of features - so they take fewer embedding dimensions to adequately reconstruct. We have added further explanations to the captions of Figures S1a and S3a that clarify this point.*

Reviewer #2:

Overall this paper addresses an important need in the field of metabolic profiling: cross dataset comparison and imputation of missing data. This work is likely to advance the field substantially. The methods seem adequate, even though not completely novel (see below):

The proposed methodology is comprised of the following pillars:

- Samples are normalized within each dataset first with a sample specific normalization factor that takes into account the proportion of missing values. Then each metabolite is converted to ranks across samples (within a dataset), with a minimum rank determined for missing values.

- Then the datasets are combined together; there are metabolites reported in one dataset but not in another and these are imputed. Non-negative matrix factorization is used with a MSE error loss function where missing variables are dropped. This gives a low dimensional embedding for all metabolites including the unmeasured ones - which can now be estimated.

In further experiments the Authors hold out various proportions of metabolites from datasets and then compare the predicted rank to the true rank. The optimal embedding size is estimates from cross validation (typically dim=30). Results show that imputed metabolites maintain normal vs tumor differential expression ranking with another dataset with known data. Finally a leave-one-dataset-out cross study validation is performed (figure 3).

Some concerns should be addressed by the Authors:

A google search reveals this paper: https://www.ncbi.nlm.nih.gov/pmc/articles/PMC8510447 "NMF-Based Approach for Missing Values Imputation of Mass Spectrometry Metabolomics Data". From a cursory look, the approach appears to be similar, apart from the ranking method applied prior to NMF. A comparison is needed to differentiate this method from the proposed one and possibly a performance comparison should be entertained.

*Reply: Thank you for this comment. We agree that our literature review should encompass the paper you suggested (Xu et al., Molecules 2021) as well as others we have found. This is clarified on Page 2 of the Introduction. However, while both MIRTH and the cited paper use NMF, their underlying methods are quite different. Most importantly, all prior related works that impute metabolomics data address a fundamentally different problem: missingness within a single batch. Our method is distinct in that we resolve the open problems of (i) how to combine multiple batches of non-overlapping data together and (ii) how to impute metabolites which were never seen in an entire batch. The rank transformation technique of MIRTH is a simple but effective approach to harmonizing across batches to solve both of these challenges. For left-censoring or other within-batch missingness, we could draw on the more sophisticated methods that have been developed for this type of missing data imputation; we have added a note on this to Page 9 of the Discussion.*

*At a more detailed level, to find the optimal number of embedding dimensions k, the above-mentioned method uses a weighted average of many NMF reconstructions of the same matrix; matrices are weighted by an improper exponential prior, which is biased toward smaller k. In contrast, MIRTH employs a dataset-wise v-fold cross-validation, simulating entirely-missing features across datasets, to find the optimal number of embedding dimensions. Additionally, in the previous work, the authors use the word “rank” to refer to the linear algebra term relating to the number of nonsingular values in the eigendecomposition of a matrix. In contrast, we use the word “rank” in the context of rank-transformation, the process by which MIRTH maps raw ion counts to their order statistics (explained on Page 11 of the Methods).*

Some of the resulting figures are not very clearly explained. For example, in figure 2c are the metabolites (or metabolite x dataset trial combinations?) ranked on the x-axis by proportion of missing samples? The caption says "as number of samples increased along the x-axis the proportion of well-predicted metabolites in a batch increased as well". This requires clarification.

*Reply: Thank you for bringing this to our attention. In the case of Figure 2c, each bar represents a metabolite measured in the dataset corresponding to the plot’s facet, where the y-axis corresponds to the median imputation performance (ρ) of that metabolite. Metabolites are ordered from high to low imputation performance - we have updated the captions for Figures 2c and 3d with this clarification. The phrase “as dataset size (number of samples) increases along the x-axis, the proportion of well-predicted metabolites in a batch increased as well” refers instead to Figure 2d, where the proportion of well-predicted metabolites for within-dataset imputation (defined as metabolites predicted with significant positive correlations with the true ranks in greater that 90% of trials) is plotted against the sample size of the dataset to illustrate the relationship between the number of training samples and imputation performance. We have added this latter note to the caption for Figure 2d.*

Also in the predicted vs true rank comparisons (e.g. figure 2f), it is not clear what each point actually represents? If each point is a trial run with different proportions of metabolites simulated as missing, this should be clearly indicated.

*Reply: Each point on the scatterplots of predicted vs. true ranks (Figures 2f, 3f, and 5c) represents one sample where the metabolite was measured. In Figures 2f and 3f, a point’s color denotes the dataset to which that sample belongs. We have updated the captions of Figures 2f, 3f, and 5c with this clarification.*

Page 10, section 4.3: "comparisons of the abundance of two metabolites in the same sample... are inadmissible". I assume this is without appropriate pre-processing but additional explanation is again required.

*Reply: LC/MS relative abundance measurements depend not only on a metabolite’s true concentration in the sample, but also on chemical properties unique to the metabolite in question. As a result, the ion counts of two different metabolite species cannot be directly compared. This is a fundamental limitation of MS metabolomics data. We have clarified this point on Page 11 of the Methods.*

Also, it is briefly mentioned in section 2.3 that batch effects within each dataset can affect the results. Stating that PCa dataset revealed 3 batches is fine but the method doesn't seem to account for this. Obviously, this is important as it will affect the ranks significantly.

*Reply: The diagnostic approach mentioned on Page 6 of the Results was useful as a way to catch potential latent batch effects, such as 3 batches being erroneously classified as a single batch. However, we cannot confirm that the PrCa dataset consisted of three sub-batches, as we are not the original data producers.*

*The point is appreciated, though, that there may be more subtle effects where a single batch has some issue that does not explicitly separate samples but does affect ranks. However, we consider this case implausible as motivated by the successful history of within-batch correction approaches. In future work, MIRTH may be generalized to handle potential within-dataset batch effects such as this one.*

*We thank the reviewer for noting this issue. We have added additional clarification on how rank-transformation aids comparison of metabolites within and across batches to Page 11 of the Methods, as well as a point of clarification on Page 8 of the discussion. The key point we make on Page 11 is that, under general assumptions of the functional form the batch effect takes, ranks are invariant to local batch effects.*

Some of the linear combinations derived to predict some key metabolites should be shown. Are the linear combinations obtained from the same metabolites in different datasets that are widely different? Are there key metabolites that are highly important for predicting many of the missing ones? i.e. Some biological interpretation of the results would be of great relevance.

*Reply: To clarify, MIRTH does not build linear functions of present metabolites to predict missing metabolites. Instead, each entry in the imputed matrix is a linear combination of an embedding vector from the sample matrix and an embedding vector from the feature matrix. These embedding matrices are generated in MIRTH’s matrix factorization step - we have included example embedding matrices as an additional Supplementary File.*

*To ascertain whether certain metabolites are highly important for predicting the missing ones, we conducted an active learning experiment to identify metabolite features most useful to predicting missing ones. The feature selection algorithm is detailed on Page 21 of the Supplementary Information. Of the 50 metabolites we identified, there were 12 lipids, 8 xenobiotics (6 of which are drugs), 6 peptides, 4 amino acids, 3 cofactors and vitamins, 1 carbohydrate, 1 nucleotide, and 15 uncharacterized metabolites. The first of these 50 metabolites, phosphoethanolamine, is involved in phospholipid metabolism. The relatively large number of drug features also raises the possibility that these play an outsized role in shaping the greater metabolome. A list of the 50 metabolites is found in Table S7, and their potential relevance to future metabolomic profiling techniques is noted on Page 8 of the Discussion.*

**Second round of review**

**Reviewer 1**

The authors have addressed all my comments satisfactorily. I believe this is a well performed study of broad interest to the community.

**Reviewer 2**

The authors have answered my queries satisfactorily.
